# Integrated single-cell and bulk transcriptomic analyses unveil a necroptosis-related prognostic model and its association with tumor microenvironment remodeling in gastric cancer

**DOI:** 10.1007/s12672-026-05109-7

**Published:** 2026-05-16

**Authors:** Yuanshuai Li, Wenting Pan, Xiaomin Ying, Xinlong Yan, Shuofeng Hu

**Affiliations:** 1 Beijing Key Laboratory of Environmental and Viral Oncology, College of Chemistry and Life Science, Beijing University of Technology, Beijing, China; 2 Center for Computational Biology, Beijing Institute of Basic Medical Sciences, Beijing, China

**Keywords:** Gastric cancer, Necroptosis, ScRNA-seq, Tumor microenvironment, Prognosis

## Abstract

**Background:**

Necroptosis has emerged as a critical regulator in tumor progression and therapeutic response, yet its prognostic significance and influence on the tumor microenvironment (TME) in gastric cancer (GC) remain poorly characterized. Therefore, it is needed to unveil the intricate relationship between GC and necroptosis.

**Methods:**

We conducted an integrative analysis of bulk and single-cell data to delineate the prognostic landscape of necroptosis in GC. A necroptosis-related (NR) model was established using transcriptomic data from the TCGA cohort and validated in an external GEO cohort. To translate this model into clinical practice, a prognostic nomogram incorporating NR scores and clinical parameters was constructed. The molecular mechanisms underlying NR variations were investigated through stemness analysis, pathway enrichment, immune cell infiltration profiling, and intercellular communication networks.

**Results:**

Consensus clustering analysis revealed pronounced heterogeneity in GC by stratifying patients into two survival-associated NR clusters (cluster 1 and cluster 2) with differences in TME characteristics. Employing machine learning approaches, a NR model was developed and validated, which demonstrated predictive ability for survival outcomes. Meanwhile, the nomogram demonstrated clinically relevant predictive accuracy, with time-dependent area under curve (AUC) values of 0.701 (1-year), 0.715 (3-year), and 0.753 (5-year). Notably, this NR model enabled robust stratification of GC patients into two distinct subgroups, both in bulk and single-cell datasets. Single-cell resolution analysis revealed patients with varying NR scores were characterized by aberrant enrichment of endothelial cells, epithelial cells, and fibroblasts, highlighting the profound impact of necroptosis on cellular composition within the TME.

**Conclusions:**

Our novel NR model deciphers the dynamic interplay between necroptosis and TME remodeling in GC, enabling robust patient stratification across both bulk and single-cell data and revealing a necroptosis-driven environment characterized by aberrant expansion of endothelial cells, epithelial cells, and fibroblasts. The clinically applicable nomogram offers a precision medicine framework for risk assessment.

**Supplementary Information:**

The online version contains supplementary material available at 10.1007/s12672-026-05109-7.

## Background

Gastric cancer (GC), the fifth most prevalent malignancy globally and the third leading cause of cancer-related deaths, presents critical clinical challenges due to delayed diagnosis and therapeutic heterogeneity [1–3]. While the 5-year survival rate for early-stage GC patients exceeds 90%, over 70% of cases are diagnosed at advanced stages with survival plummeting to 18–50% [4]. Furthermore, the heterogeneous tumor microenvironment (TME) contributes to variable therapeutic responses [1, 3, 5]. Current prognostic models are limited by their inability to adequately capture the complexity of the TME, highlighting the urgent need to identify novel prognostic biomarkers and therapeutic targets to enable precision medicine approaches.

Necroptosis, a programmed cell death pathway regulated by RIP1, RIP3, and MLKL, exhibits dual roles in tumorigenesis [6]. On the one hand, cancer cells undergoing necroptosis release damage-associated molecular patterns (DAMPs) and tumor-specific antigens, which serve as potent adjuvants that recruit and activate effector immune cells. This process can potentiate anti-tumor immunity and suppress tumor progression. On the other hand, necroptosis induction triggers the secretion of interleukin-1 alpha from dying tumor cells. This cytokine acts as a master regulator that reprograms myeloid cells toward an immunosuppressive phenotype within the TME, thereby limiting the efficacy of both conventional chemotherapy and combinatorial regimens with immunotherapy [7, 8]. Given the complex role of necroptosis in tumor progression, precise modulation of the necroptosis pathway (inhibition or activation) is considered a promising strategy for therapy.

The advent of single-cell RNA sequencing (scRNA-seq) has revolutionized the characterization of TME heterogeneity, enabling high-resolution profiling of cellular diversity [9–11]. At the single-cell level, the examination of tumor tissues facilitates precise identification of distinct cell populations and potential therapeutic targets, thereby deepening our understanding of tumor dynamics and improving patient survival outcomes [12–14]. However, the molecular mechanisms of necroptosis in GC remain poorly understood. While previous studies have developed necroptosis-related prognostic models for GC using bulk transcriptomic data [15, 16], these approaches are inherently limited by their inability to deconvolve cellular heterogeneity. Consequently, they fail to identify the specific cellular compartments or the complex changes in intercellular communication networks within the necroptosis-influenced TME.

To overcome these limitations, we integrated single-cell RNA sequencing with bulk transcriptomic data for the first time. Through multi-omics analysis, we systematically characterized immune infiltration dynamics, stemness features, enriched pathway patterns, and intercellular communication networks across patients with distinct NR scores. Furthermore, we developed an NR-based nomogram, which serves as a clinically actionable tool for survival prediction and personalized treatment optimization. In summary, this study provides a comprehensive analysis of necroptosis in GC at the single-cell resolution, fundamentally advancing our understanding of necroptosis-mediated TME regulation. It overcomes limitations of previous bulk-level studies (masking cellular heterogeneity), providing a mechanistic framework for precision oncology strategies targeting necroptosis.

## Methods

### Data acquisition

The scRNA-seq dataset of GC samples was downloaded from the Gene Expression Omnibus (GEO) database (http://www.ncbi.nlm.nih.gov/geo/; GSE183904), including 26 tumor patients**.** The bulk RNA-seq data, clinical information (age, sex, TNM stage, survival outcomes), and somatic mutation data for stomach adenocarcinoma were obtained from The Cancer Genome Atlas (TCGA; https://portal.gdc.cancer.gov/) and GEO database (GSE84437 and GSE26942). Tumor mutational burden (TMB) was calculated using the “maftools” R package. All datasets analyzed in this study were obtained from publicly available sources.

### Identification of NR genes and tumor classification

A list of 159 necroptosis-related genes (NRGs; Table S1) was obtained from the KEGG (http://www.genome.jp/kegg/) and The Molecular Signatures Database (MSigDB) (http://www.gseamsigdb.org/gsea/msigdb/) databases [17]. According to the univariate Cox regression analysis, the NRGs associated with the overall survival (OS) were selected. Consensus clustering was performed on TCGA-GC samples using the “ConsensusClusterPlus” R package, based on the expression levels of the OS-related NRGs. Protein–protein interaction (PPI) networks were constructed using the Search Tool for the Retrieval of Interacting Genes (STRING; https://string-db.org/) database, and the copy number variation (CNV) landscapes were visualized with the “RCircos” R package.

### Evaluation of infiltrating immune cells

In bulk RNA-seq data, to quantify immune and stromal cell infiltration, the ESTIMATE R package was employed to calculate immune and stromal scores. Meanwhile, the relative proportions of 22 immune cell types were estimated using the “CIBERSORT” R package. Immune pathway activity was assessed via single sample gene set enrichment analysis (ssGSEA) implemented in the “GSVA” R package, utilizing the “LM22” gene signature [18].

### Construction and validation of NR prognostic model

Differentially expressed genes (DEGs) between distinct NR tumor clusters, as well as between normal and tumor tissues were identified utilizing the “limma” R package with a criteria of false discovery rate (FDR) ≤ 0.05 and |log_2_ Fold Change (FC) |≥ 1. The overlapping DEGs common to both comparisons were selected for model construction. GC patients in the TCGA database were designated as the training cohort, and samples in the GEO database (GSE84437) were served as the validation set. To select optimal features from the intersecting genes, we employed a combination of 101 machine learning method configurations spanning 10 algorithms [19, 20]. These algorithms included least absolute shrinkage and selection operator (LASSO), supervised principal components (SuperPC), elastic network (Enet), stepwise Cox, gradient boosting machine (GBM), random forest (RSF), partial least squares regression for Cox (plsRcox), survival support vector machine (Survival-SVM), CoxBoost, and ridge regression. Subsequently, the NR prognostic model was constructed in the training set. Utilizing the formula derived from the training cohort, NR scores were calculated for both cohorts. Patients were divided into low- and high-NR groups based on the median score. Kaplan–Meier (KM) survival curve analysis was used to compare the OS, disease free interval (DFI), progression free interval (PFI), and disease specific survival (DSS) time between the two NR groups. The time-dependent ROC curve was applied to assess the sensitivity and specificity of the prognostic model, utilizing the “survivalROC” R package.

At the single-cell level, the NR scores for individual cells were calculated using the computational formula and model genes obtained from bulk RNA-seq data. Subsequently, an average NR score was calculated for each patient sample by aggregating the scores of all cells from that sample. Patients were then stratified into NR^high^ and NR^low^ groups based on the median of these average sample scores.

### Establishment of prognostic nomogram

A nomogram integrating the NR score and clinical factors was constructed using the “rms” R package to predict the OS of GC patients. The predictive performance of the nomogram was evaluated through the concordance index (C-index), receiver operating characteristic (ROC) curves, calibration plots, and decision curve analysis (DCA).

### Functional enrichment analysis

DEGs between the two groups were identified to explore differences in functional pathways. Gene Ontology (GO), and Kyoto Encyclopedia of Genes and Genomes (KEGG) analyses were performed on the DEGs using the “ClusterProfiler” R package. Additionally, Gene Set Enrichment Analysis (GSEA) and Gene Set Variation Analysis (GSVA) were conducted in both groups with default parameters, based on the 50 hallmark gene sets (h.all.v2024.1.Hs.symbols.gmt) from the MSigDB database (https://www.gsea-msigdb.org/gsea/msigdb/index.jsp).

### Preprocessing of single-cell RNA-Seq data

Single-cell RNA-seq data were processed taking the “Seurat” R package (version 4.3.0.1). Potential doublets were identified and removed with the “DoubletFinder” R package. DoubletFinder was applied to each sample independently to identify and remove potential doublets. Following the standard workflow, artificial doublets were generated with the proportion set to 0.25, and the optimal proportion of artificial nearest neighbors was selected based on the BCmetric derived from the sweep analysis. The expected doublet rate was estimated according to the number of cells (8 per 1000 cells). The proportion of homotypic doublets was estimated from the initial clustering results to adjust the expected number of doublets. Cells classified as high-confidence doublets were subsequently excluded from downstream analysis. Then, the low-quality cells were filtered based on the following thresholds: (1) cells with gene counts more than 98% or fewer than 2% of the total distribution, and (2) cells with over 20% of reads aligned to the mitochondrial genome. The remaining cells were normalized (via “NormalizeData” function), and highly variable genes (HVGs) were identified (“FindVariableFeatures”). Scaled data (“ScaleData”) underwent principal component analysis (PCA; “RunPCA”). Batch effects were corrected using the “harmony” algorithm. Dimensionality reduction was performed using uniform manifold approximation and projection (UMAP) and t-distributed stochastic neighbor embedding (t-SNE) based on the top 30 principal components. Cell clusters were resolved taking the “FindNeighbors” and “FindClusters” functions.

### Annotation of cell clusters

According to the “FindAllMarker” method with recommend parameters, the DEGs of each subcluster were obtained. Cell clusters were annotated based on DEGs and canonical markers that including B cells (CD79A, MZB1, and IGHA2), T/innate lymphoid cells (ILCs; CD3D, CD3G, and NKG7), epithelial cells (CDH1, KRT19, and EPCAM), fibroblasts (DCN, COL1A1, and THY1), myeloid cells (S100A9, CD14, and CD68), and endothelial cells (PECAM1, CLDN5, and ENG).

### Estimation of single-cell copy number variation

To distinguish malignant cells from non-malignant epithelial cells, “inferCNV” R package (https://github.com/broadinstitute/inferCNV) was used to estimate the copy number variations (CNVs) [21]. Myeloid cells and T/ILCs were selected as reference populations. The analysis was performed with Hidden Markov Model (HMM)-based smoothing disabled (HMM = FALSE) and denoising enabled (denoise = TRUE).

### Functional annotation scoring

To evaluate the functional states of cell types across different NR groups, a set of literature-supported functional genes were curated (Table S2). Furthermore, stemness-related genes from CancerSEA database (http://biocc.hrbmu.edu.cn/CancerSEA/) [21, 22] were obtained. In scRNA-seq data, functional signatures were quantified using the “AddModuleScore” algorithm and “AUCell” R package [23]. For bulk RNA-seq data, functional activities were assessed with “ssGSEA”.

Cell-type-specific markers were identified through differential expression analysis via “FindAllMarkers” function in the “Seurat” R package. Genes were ranked by average log_2_(FC) in expression, and the top 50 markers for each cell type were selected as a signature gene set. These signatures were subsequently quantified in bulk RNA-seq data using “ssGSEA” to facilitate survival analysis.

### Intercellular communication analysis across NR groups

Cell–cell communication patterns among different NR groups were inferred with “CellChat” R package (version 1.1.0) [24]. With the recommended parameters, cell–cell interaction networks were quantified and compared across NR groups. Finally, differentially communicated networks were visualized using the “netVisual_diffInteraction” function.

### Statistical analysis

All statistical analyses were performed utilizing R (v4.3.1), python (v3.11.4) and Linux (CentOS 7.9). Survival differences for genes were assessed using the log-rank test, with optimal low/high cutoffs determined via “surv_cutpoint” function. Group comparisons were performed using the Wilcoxon signed-rank test. Statistical significance was defined as *p* ≤ 0.05. The analytical workflow is summarized in Figure S1.

## Results

### Associations between necroptosis and clinical characteristics

**Fig. 1:**
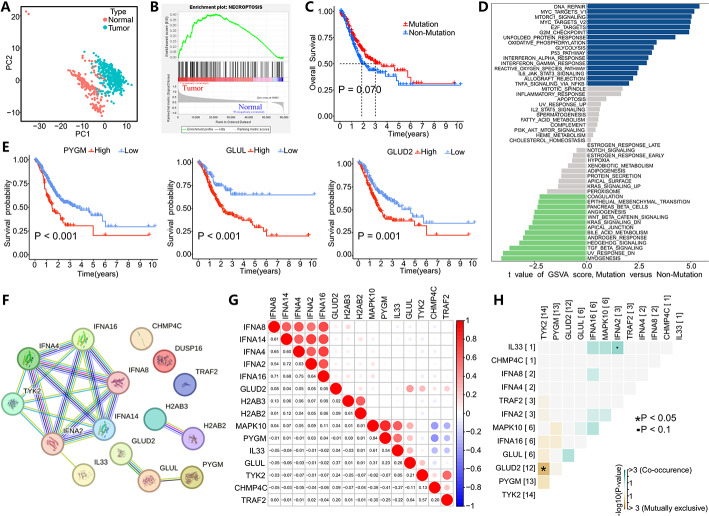
Characteristics of NR genes in GC samples from the TCGA-STAD cohort. **A** Scatter plot of PCA scores comparing tumor and normal samples based on NR genes. **B** The GSEA results based on NR genes. **C** The KM analysis comparing OS between necroptosis associated mutation and non-mutation groups. **D** The GSVA comparing mutant and non-mutant groups using 50 hallmark gene sets. **E** Survival analyses of the OS-related genes between low- and high-expression groups, using optional cut-off values. **F** PPI network showing the interactions among the OS-related genes. **G** Heatmap displaying the correlation among OS-related genes in tumor samples using spearman correlation analysis. **H** Correlation analysis of the mutations in OS-related genes

We first performed dimensionality reduction on the TCGA-STAD cohort to investigate the relationship between NR pathways and tumorigenesis. Based on 159 NR genes, PCA result showed clear segregation between tumor and normal samples (Fig. 1A). GSEA analysis revealed significant enrichment of necroptosis gene set in tumor samples (Fig. 1B).

Next, we characterized the mutation landscape of 159 NR genes in across 431 GC patients and identified mutations in 136 cases (31.55%) (Fig. S2A). Missense mutations represented the predominant type of all mutations (Fig. S2B). Notably, we found that the mutation group showed a trend toward prolonged survival outcomes (Fig. 1C). The GSVA results revealed significant enrichment of NR pathways, such as TNFA signaling via NFKB pathway and reactive oxygen species pathway in tumors with NR gene mutations (Fig. 1D). These results suggested that mutation of NR genes induce the alteration of activated pathways in tumors, which may contribute to the outcomes of GC patients.

To identify NR genes with prognostic significance for OS, we conducted a univariate Cox regression analysis and identified 15 survival-related genes. Among these genes, TRAF2, CHMP4C, and TYK2 were associated with favorable prognosis, while the remain genes indicated poor prognosis (Table S3). The survival curves exhibited similar result (Fig. 1E; Fig. S2C). Furthermore, the PPI network in the STRING database identified IFNA2, IFNA4, IFNA8, IFNA14, IFNA16, and TYK2 as hub genes, based on their high degree centrality (Fig. 1F), with their co-expression relationships further validated by correlation analysis (Fig. 1G). In addition, significant co-occurrence patterns were observed between IFNA2 and IL33, while GLUD2 and TYK2 exhibited mutually exclusive patterns (Fig. 1H), suggesting diverse genomic alterations.

The “RCircos” R package was employed to map locations of survival-related genes across human chromosomes (Fig. S2D). However, we were unable to locate information for H2AB3 and H2AB2 in the position file. Seven genes mainly displayed copy number deletions, whereas six genes exhibited amplifications (Fig. S2E). Tumor samples showed higher expression of CHMP4C, GLUD2, TRAF2, TYK2, and H2AB2, but lower levels of MAPK10, IL33, and PYGM compared to normal tissues (Fig. S2F). Overall, this expression pattern broadly aligns with copy number amplifications and deletions, suggesting that copy number variations may promote aberrant expression of OS-related genes through dosage effects, potentially contributing to GC progression.

### Dissection of GC heterogeneity based on survival-related NR genes

**Fig. 2:**
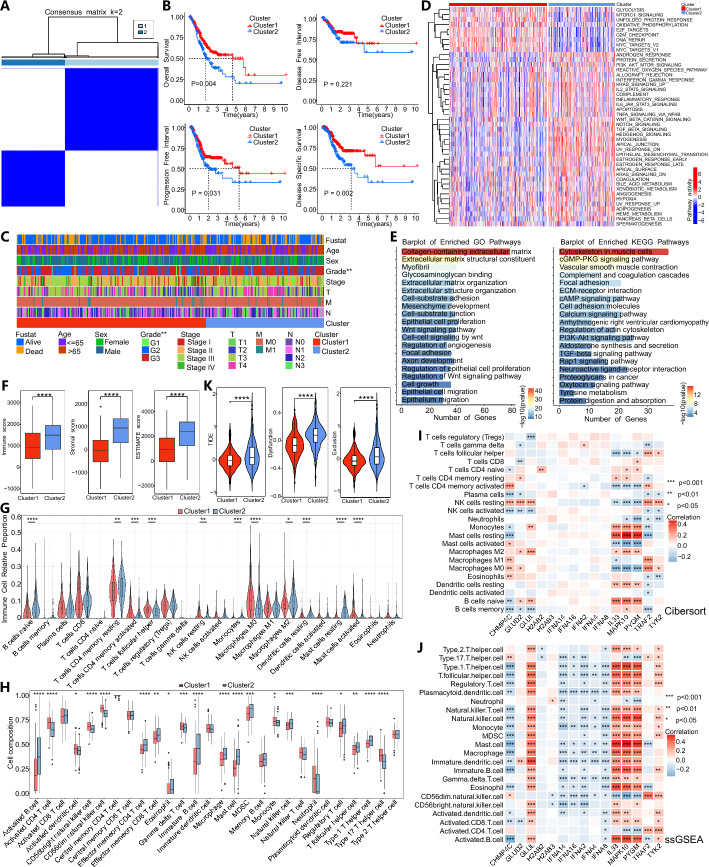
Clinical association and immune landscape of tumor subtypes. **A** Consensus clustering matrix for clustering variable (k) = 2. **B** The KM survival curves comparing OS, DFI PFI, and DSS between different clusters. **C** Heatmap illustrating the correlations between tumor clusters and clinical features. **D** Heatmap displaying the characteristics of hallmark gene sets across various clusters using GSVA. **E** The GO and KEGG annotation of DEGs between the two tumor clusters. **F** Comparative analysis of immune scores, stromal scores, and ESTIMATE scores between tumor clusters. **G** Analysis of immune cell infiltration patterns across 22 immune cell subsets calculated by the “CIBERSORT” R algorithm. **H** Evaluation of the infiltration of immune cells and immune-related pathways using the “ssGSEA” R function in the two tumor clusters. **I**, **J** Heatmap displaying correlations between OS-related regulators and immune cells (**I**), as well as immune-related pathways using spearman correlation analysis (**J**). **K** The TIDE scores, exclusion scores, and dysfunction scores across the two tumor clusters. (*P < 0.05, **P < 0.01, ***P < 0.001, ****P < 0.0001)

Leveraging the 15 survival-related NR genes, we sought to characterize GC subclusters. Consensus clustering analysis stratified the patients into two distinct clusters (Fig. 2A; Fig. S3A, B). There were 244 GC patients in the cluster 1, and 166 patients in the cluster 2. The t-SNE analysis revealed clear separations among patients between these two clusters (Fig S3C). Cluster 1 showed prolonged OS (*P* = 0.004), PFI (*P* = 0.031) and DSS (*P* = 0.002) comparing with cluster 2, though no significant difference was observed in DFI (*P* = 0.221; Fig. 2B). Furthermore, a significant correlation was observed between the two clusters and tumor grades among clinical features (Fig. 2C). The GSVA indicated that cluster 1 exhibited enrichment in glycolysis, G2M checkpoint, and DNA repair pathways, while cluster 2 was enriched in WNT/β-catenin, hypoxia, TNFA signaling via NFKB, and TGF-beta signaling pathways (Fig. 2D). It suggests that the co-enrichment of TNFA signaling via NFKB and hypoxia pathways indicates potential activation of necroptosis, while WNT/β-catenin and TGF-beta signaling may serve as regulatory modulators of the cell death pathway in cluster 2. Between the two tumor clusters, we identified 585 DEGs. Functional enrichment analysis showed that the DEGs were involved in NR pathways, such as the calcium signaling pathway, PI3K-Akt signaling pathway, and regulation of the actin cytoskeleton (Fig. 2E).

To characterize the immune landscape, ESTIMATE algorithm was applied to assess differences between the two clusters. The results exhibited significantly higher immune (*P* < 0.001), stromal (*P* < 0.001), and ESTIMATE scores (*P* < 0.001) in cluster 2 compared to cluster1 (Fig. 2F). We further explored infiltration of various immune cell types using CIBERSORT and ssGSEA [25]. The CIBERSORT analysis identified significantly higher infiltration of naive B cells, resting memory CD4 T cells, M1 macrophages, and resting dendritic cells in cluster 2 (Fig. 2G). The ssGSEA result showed higher enrichment of B cells, mast cells, and NK cells in cluster 2 (Fig. 2H). We further explore the expression patterns of various immune-related markers. Cluster 2 exhibited higher expression levels of immune checkpoint genes (TIGIT, HAVCR2, and BTLA) compared to cluster 1, whereas the expression of CTLA4, PDCD1, and LAG3 remained comparable between clusters. Additionally, T cell markers (CD3G, CD8A, and CD4) and M2 markers (CD209, CD163, and CD86) were significantly up-regulated in cluster 2. Notably, both cytotoxic genes (GZMK, NKG7, and IL2) and resident genes (RUNX3, CD69, and NR4A3) were also substantially elevated in cluster 2 (Fig. S3D). Most of the 15 survival-related NR genes were significantly correlated with the infiltration of immune cell types, especially mast cells (Fig. 2I, J), a finding confirmed in independent GEO datasets (Fig. S3E, F). H2AB2 and H2AB3 were not found in these datasets. Collectively, the cluster 2 represented a subgroup characterized by a complex immune microenvironment and features of immune exhaustion. This finding suggested a critical role of cell necroptosis in TME alteration, which may augment immunogenicity.

Thus, we conducted Tumor Immune Dysfunction and Exclusion (TIDE) analysis to predict responses to immunotherapy. Our analysis revealed that cluster 2 exhibited significantly higher TIDE, dysfunction, and exclusion scores (Fig. 2K). In line with these findings, cluster 2 showed a lower proportion of immunotherapy sensitive samples (Fig. S3G). Collectively, the NR-based classification, though requiring mechanistic validation, demonstrates potential as a stratification tool for GC immunotherapy trials.

### Development and prognostic analysis of a NR prognostic model of GC

**Fig. 3:**
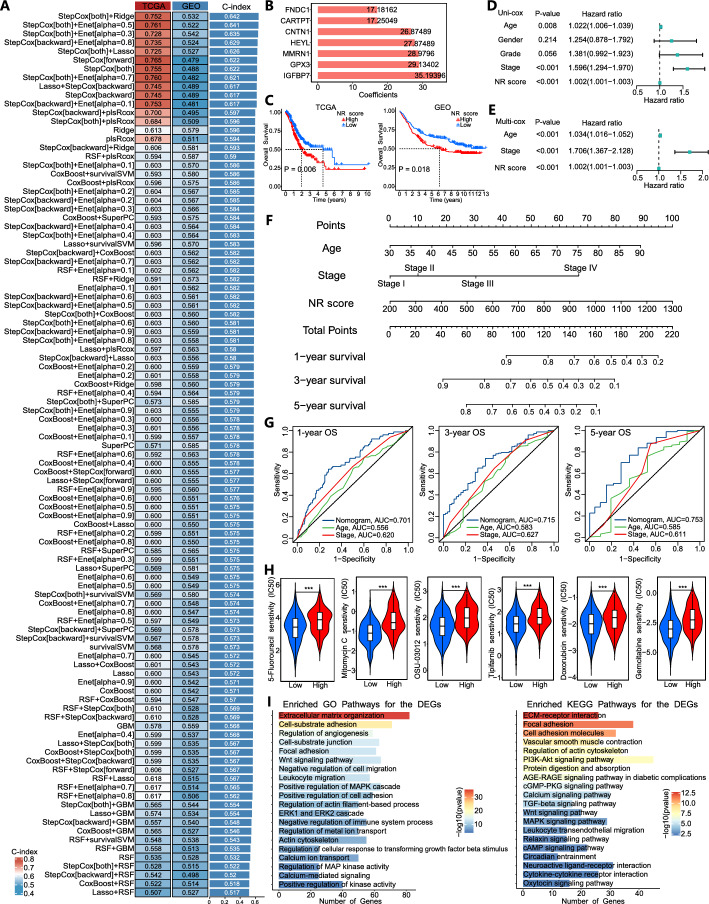
Construction of the NR prognostic model for GC. **A** Machine learning was utilized to develop an NR model, with a C-index across 101 cancer models from the TCGA and GSE84437 datasets. **B** Profiles of gene coefficient in the prognostic model were illustrated. **C** Survival analyses comparing the OS of GC patients in distinct NR groups were performed in both the training (TCGA) and test (GSE84437) cohorts. **D, E** Univariate (**D**) and multivariate (**E**) Cox regression analyses of risk scores and various clinical parameters in the training cohort. **F** Nomogram integrating NR scores and clinical features was developed to predict 1-, 3- and 5-year OS in the TCGA dataset. **G** ROC curve analysis of the nomogram in predicting the OS. **H** Violin plots showing IC50 distributions for drugs with significantly reduced sensitivity in NR^high^ group. **I** Identification of significantly enriched pathways through GO analysis and KEGG analysis of DEGs between the distinct NR subgroups

To develop a NR prognostic model for GC patients, we conducted a systematic screening of candidate genes. 3,448 DEGs between tumor and normal tissues, and 585 DEGs between cluster 1 and cluster 2 were identified. Intersection of these DEGs yielded 179 overlapping genes that were selected for further prognostic modeling. These candidates underwent different combination of various machine learning analysis, and the C-index of each model was calculated in both training and test cohorts (Fig. 3A). Considering the gene count and C-index in each modal, we selected the “CoxBoost” and “plsRCox” algorithms for prognostic gene screening. Finally, a seven-gene model with robust prognostic performance was identified (MMRN1, IGFBP7, CNTN1, HEYL, CARTPT, GPX3, and FNDC1) (Fig. 3B). Survival analysis revealed significant associations between the model genes and OS (Fig. S4A).

Based on these seven NR genes, a prognostic model was developed leveraging gene expression and regression coefficients. The GC samples were stratified into two groups based on the median of NR risk scores. PCA and t-SNE analysis showed clear separation between the two groups (Fig. S4B, C). KM survival analysis indicated that GC patients with high NR risk scores exhibited significantly poorer prognosis in both TCGA and GEO cohorts (Fig. 3C). Remarkably, the NR risk scores presented a progressive increase across advanced pathological stages and T stages, demonstrating their association with disease severity (Fig. S4D).

Next, we systematically assessed the predictive performance of the NR risk model to evaluate its clinical utility. While the NR model showed robust performance in the TCGA training cohort (1-year AUC: 0.613, 3-year: 0.643, 5-year: 0.719), its performance in the GEO validation cohort was moderate (1-year AUC: 0.557, 3-year: 0.596, 5-year: 0.617) (Fig. S4E). This variation may be attributed to inherent heterogeneity between cohorts, including differences in sample collection, sequencing platforms, and patient demographics. Both univariate and multivariate Cox regression analyses in the training cohort indicated that the NR model was an independent prognostic biomarker compared to other clinical features, suggesting its independent predictive capability for patient outcomes (Fig. 3D, E). We developed a comprehensive nomogram integrating independent clinical features from multivariate Cox regression to estimate 1-, 3-, and 5-year survival probabilities for GC patients (Fig. 3F). The calibration curve analysis validated the nomogram’s high predictive accuracy (Fig. S4F). Notably, time-dependent ROC analysis and DCA revealed that the nomogram exhibited moderate potential benefit compared to other clinical characteristics for OS, achieving AUC values of 0.701, 0.715 and 0.753 for 1-year, 3-year, and 5-year predictions, respectively (Fig. 3G; Fig. S4G). We then evaluated the association of the NR risk model with chemotherapeutic efficacy. We predicted half-maximal inhibitory concentration (IC50) values for GC patients in the TCGA cohort. NR^high^ patients presented high IC50 scores for mitomycin C, doxorubicin, and gemcitabine (Fig. 3H), while NR^low^ patients presented high IC50 scores for midostaurin and PHA-665752 (Fig. S4H). These findings suggest that NR scores may serve as potential predictive indicators for chemotherapy sensitivity.

To elucidate functional differences between NR groups, we performed enrichment analysis for the DEGs. Results showed that the NR^high^ group was characterized by activation of extracellular matrix (ECM) signaling pathways, including ECM organization, cell-substrate adhesion, and junction pathways. Additionally, this group exhibited significant enrichment in tumorigenic pathways such as PI3K-Akt signaling, and calcium signaling (Fig. 3I). GSVA further indicated enhanced activity in Notch, and TGF-beta signaling pathways (Fig. S4I). Collectively, these findings highlight that the NR model is associated with remodeling the stromal microenvironment, characterized by ECM dysregulation. The enrichment of PI3K-Akt, and TGF-beta pathways further suggests a crosstalk between tumor cells and stromal components, potentially driving therapeutic resistance.

### Landscapes of cell composition in different NR groups

**Fig. 4:**
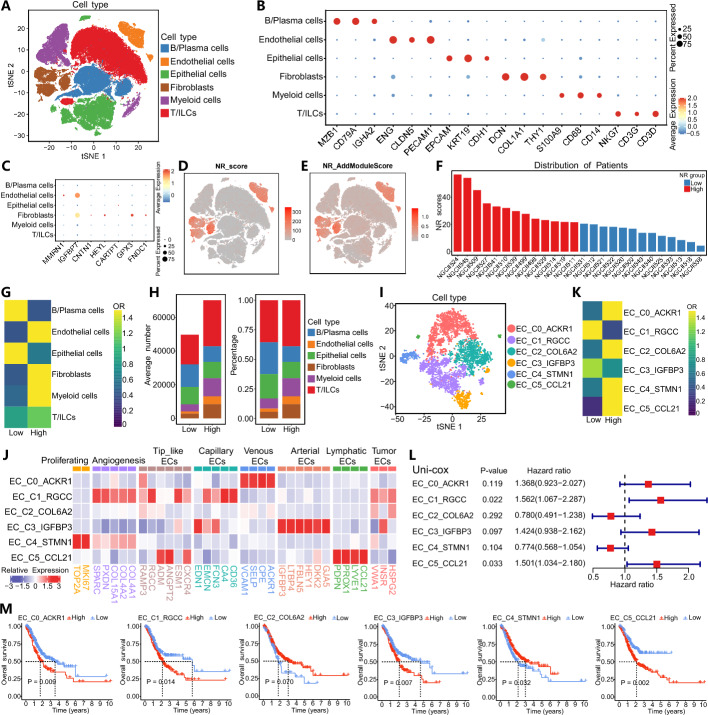
Landscape of the NR model in single-cell RNA sequencing data from GC samples. **A** The t-SNE plot displaying the annotations for cell types. **B** Dot plot illustrating classical marker genes in each cell type.** C** Dot plot displaying the average expression of model genes across various cell types. **D**, **E** The t-SNE plots depicting expression scores of model gene signatures, calculated using the scoring formula derived from bulk RNA-seq data (**D**) and the “AddModuleScore” function of the “Seurat” R package (**E**). **F** Box plot showing the distribution of NR scores in GC patients. **G** Heatmap displaying the OR of major cell types in the NR^low^ and NR^high^ status. **H** Bar plot indicating the proportions of cell types in the NR^low^ and NR^high^ groups. **I** The t-SNE plot displaying the distribution of endothelial cell (EC) subtypes. **J** Heatmap showing gene expression patterns across subtypes. **K** OR of endothelial cell subtypes in both NR status. **L** Univariate Cox regression analysis for endothelial cell subtypes in TCGA cohort. **M** The OS analysis of TCGA-GC patients grouped by endothelial cell subtypes (high/low) based on optimal cut-off

To resolve cellular heterogeneity in different NR risk groups, we utilized a scRNA-seq dataset encompassing 119,878 high-quality cells for downstream analysis (Fig. S5A). We identified fibroblasts, endothelial cells, myeloid cells, epithelial cells, B/plasma cells, and ILCs (Fig. 4A), through the expression patterns of canonical markers (Fig. 4B; Fig. S5B). Notably, the proportions of different cell types varied in different patients, highlighting the heterogeneous nature of GC (Fig. S5C).

Compared to other cell types, MMRN1 and IGFBP7 (the genes of NR model) exhibited high expression levels in endothelial cells, while CNTN1, HEYL, GPX3, and FNDC1 highly expressed in fibroblasts (Fig. 4C). We further utilized the scoring formula of NR model to calculate NR score for each cell. The results revealed that the scores were predominantly elevated in fibroblasts and endothelial cells (Fig. 4D). We got a consistent trend based on the “AddModuleScore” method (Fig. 4E). These findings suggest the critical roles of fibroblasts and endothelial cells, which provides clues for our subsequential exploration.

By calculating the NR scores based on the pooling expression of all cells in each sample, we classified the patients into NR^high^ and NR^low^ groups (Fig. 4F). The odds ratios (OR) were calculated and revealed that myeloid cells, fibroblasts and endothelial cells were predominant in the NR^high^ group (Fig. 4G) [26]. The proportion analysis further demonstrated a higher enrichment of myeloid cells, endothelial cells and fibroblasts in NR^high^ group compared to NR^low^ group (Fig. 4H). Given that fibroblasts and endothelial cells exhibited higher enrichment in the NR^high^ group, we hypothesized that these stromal components might be particularly influenced by necroptotic activity and subsequently focused our downstream analysis on characterizing their heterogeneity and functional states across NR groups.

### Diversity of endothelial cells across different NR groups

To characterize EC heterogeneity associated with necroptosis, we performed subpopulation analysis and identified six distinct subtypes (EC_C0_ACKR1, EC_C1_RGCC, EC_C2_COL6A2, EC_C3_IGFBP3, EC_C4_STMN1, and EC_C5_CCL21) (Fig. 4I; Fig. S5D). The NR gene, IGFBP7, exhibited high expression across all endothelial subtypes, while MMRN1 showed elevated expression specifically in EC_C5_CCL21 (Fig. S5E). The EC_C1_RGCC exhibited characteristics of tumor endothelial cells (tumor ECs), and the lymphatic ECs-related genes (CCL21, LYVE1, PROX1, and PDPN) were upregulated in the EC_C5_CCL21 (Fig. 4J).

The endothelial cell subtypes exhibited distinct enrichment patterns between the NR groups. The proportion of EC_C5_CCL21, a subtype related to lymphatic functions, increased in NR^high^ risk groups (Fig. 4K; Fig. S5F). Additionally, we noticed that the high EC_C5_CCL21 abundance was associated with poor survival of GC patients (Fig. 4L, M). Moreover, EC_C5_CCL21 abundance exhibited a positive correlation with the NR score (Spearman’s correlation), suggesting that this subset may promote tumor progression (Fig. S5G). These findings suggest that necroptosis may be associated with lymphangiogenesis, which in turn causes changes in the tumor microenvironment, ultimately leading to a poor prognosis in GC.

### Characteristics of fibroblasts across different NR subtypes

**Fig. 5:**
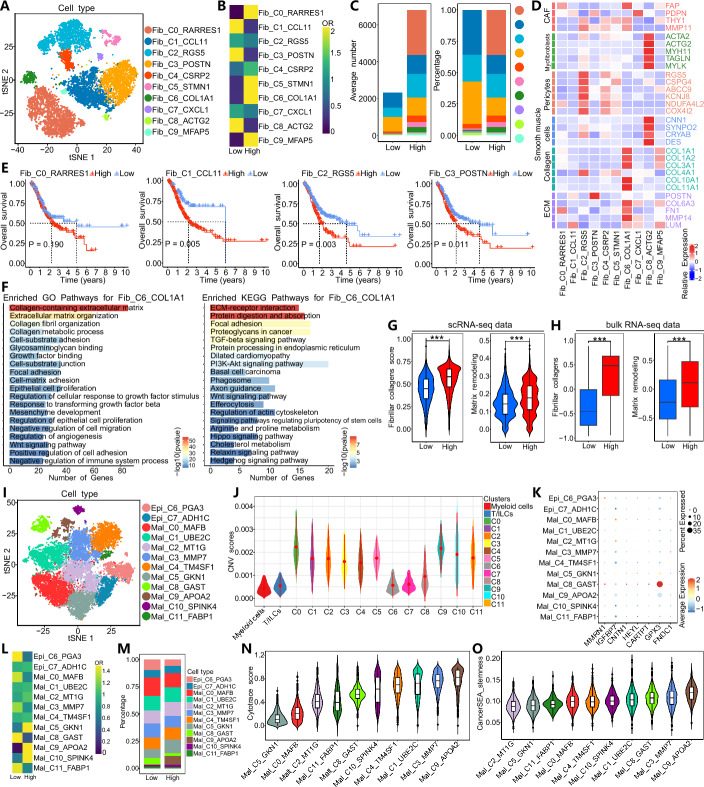
Characteristics of fibroblasts and epithelial cells in the NR model. **A** The t-SNE plot illustrating the annotations of total fibroblasts. **B** OR comparing fibroblast subtypes in the two NR status. **C** Distribution analysis of fibroblasts between NR groups. **D** Heatmap showing the expression patterns of functional genes across different fibroblast subtypes. **E** KM curve analysis displaying OS of TCGA-GC samples, stratified by cell type abundance, with optimal cutoff values determined using the “survivalROC” R package. **F** GO and KEGG pathway enrichment analysis of DEGs in Fib_C6_COL1A1. **G** Violin plot analysis of fibrillary collagen and matrix remodeling scores across fibroblasts, calculated via “AUCell” R package, between the two NR subgroups. **H** Box plots illustrating the enrichment scores of fibrillar collagens and matrix remodeling signatures across different NR groups in the TCGA cohort. **I** The t-SNE plot of epithelial cell populations from scRNA-seq data. **J** Violin plots illustrating the status of copy number alterations across epithelial cell clusters, T/ILCs, and myeloid cells.** K** Dot plot showing the expression levels of model genes.** L** Heatmap displaying the OR of epithelial subtypes between NR^low^ and NR^high^ groups. **M** Subtype composition analysis for epithelial cells across different NR groups.** N** Violin plots displaying the CytoTRACE scores across various malignant cell subtypes. **O** The stemness scores across malignant cells, calculated by “AUCell” R package based on the CancerSEA stemness signatures in scRNA-seq data

To investigate fibroblast heterogeneity linked to necroptosis in GC, we applied unsupervised clustering to 11,206 fibroblasts, revealing ten transcriptionally distinct subpopulations (Fig. 5A; Fig. S6A, B). Notable variation in cell subtypes was observed among different patients (Fig. S6C). Among the 7 key genes in NR model, elevated expression of IGFBP7, was observed in Fib_C2_RGS5, whereas BMP3 showed increased expression in Fib_C9_MFAP5 (Fig. S6D). Compared to NR^low^ group, Fib_C0_RARRES1, Fib_C5_STMN1, Fib_C6_COL1A1 and Fib_C9_MFAP5 showed increased proportions (Fig. 5B, C).

Based on gene expression analysis, we observed significant upregulation of cancer-associated fibroblasts (CAFs) markers (FAP, THY1, and MMP11), ECM-associated genes (COL6A3 and MMP14), and various collagen-genes (COL1A1, COL1A2, and COL3A1) in Fib_C6_COL1A1 (Fig. 5D). Univariate Cox regression analysis also suggested Fib_C6_COL1A1 as a poor prognostic indicator for GC patients (Fig. S6E). Additionally, KM analysis of OS revealed significant prognostic stratification among fibroblast clusters, including Fib_C1_CCL11, Fib_C2_RGS5, Fib_C3_POSTN, Fib_C4_CSRP2, Fib_C5_STMN1, Fib_C6_COL1A1, Fib_C8_ACTG2, and Fib_C9_MFAP5 (Fig. 5E; Fig. S6F). Notably, the abundance of Fib_C6_COL1A1 was also significantly positively correlated with the NR score, suggesting that this fibroblast subset may contribute to tumor progression by influencing necroptosis‑related activity within the TME (Fig. S6G).

To investigate the potential mechanisms underlying the association between Fib_C6_COL1A1 and poor patient survival, we performed gene set enrichment analysis. The results showed significant enrichment in ECM-receptor interaction, collagen-containing extracellular matrix, and collagen fibril organization pathways (Fig. 5F). These findings suggested that Fib_C6_COL1A1 played a crucial role in collagen production and fibrotic progression for NR^high^ patients.

Consistent with established roles of CAFs in tumor progression [27, 28], we identified distinct NR-dependent fibroblast polarization patterns. Fibroblasts from NR^low^ tumors were predominantly enriched in RNA processing pathways, whereas fibroblasts from NR^high^ tumors showed significantly enrichment of extracellular matrix organization, and hypoxic response pathways (Fig. S6H). Quantitative analysis revealed significantly collagen transcription (*P* < 0.001) and matrix remodeling activity (*P* < 0.001) in NR^high^ fibroblasts (Fig. 5G), with validation in bulk RNA-seq data (Fig. 5H). These results suggest that necroptotic activity is associated with fibroblast activation, characterized by collagen and matrix-related pathway upregulation, potentially contributing to tumor microenvironment remodeling in NR^high^ tumors.

### The heterogeneity of epithelial cells across different NR subtypes

Accumulating evidence shows necroptosis promotes cancer proliferation and metastasis. Given epithelial cells’ sensitivity to microenvironmental disruptions, studying necroptosis-induced changes in tumor cells is crucial. Differential analysis of intercellular communication across NR subgroups uncovered different interaction networks between epithelial cells and fibroblasts or endothelial cells, underscoring epithelial cells' pivotal role in tumor progression (Fig. S7A). We therefore systematically characterized 19,958 epithelial cells and identified 12 distinct subgroups (Fig. 5I; Fig. S7B, C). The result of inferCNV indicated that all clusters were malignant cells, with the exception of clusters 6 and 7 (Fig. 5J; Fig. S7D). GPX3, one of the model genes, was highly expressed in Mal_C8_GAST (Fig. 5K). Mal_C5_GKN1 and Mal_C9_APOA2 were predominantly enriched in NR^high^ group (Fig. 5L, M).

The CytoTRACE analysis revealed that Mal_C9_APOA2 exhibited the highest stemness score (Fig. 5N) [29]. Consistent with the above results, independent evaluation using stemness-related genes confirmed the high stemness score of Mal_C9_APOA2 (Fig. 5O). Although survival analysis demonstrated no significant prognostic association for Mal_C9_APOA2 signature in GC patients (Fig. S7E), enrichment analysis revealed that it was associated with several key pathways involved in tumor progression and microenvironment regulation, including response to hypoxia, neutrophil migration, and epithelial cell proliferation (Fig. S7F). Taken together, these findings suggest that necroptosis may influence the TME within NR^high^ group, thereby enhancing cancer stemness and promoting tumor progression and metastasis.

### Differential intercellular communications influenced by necroptosis

**Fig. 6:**
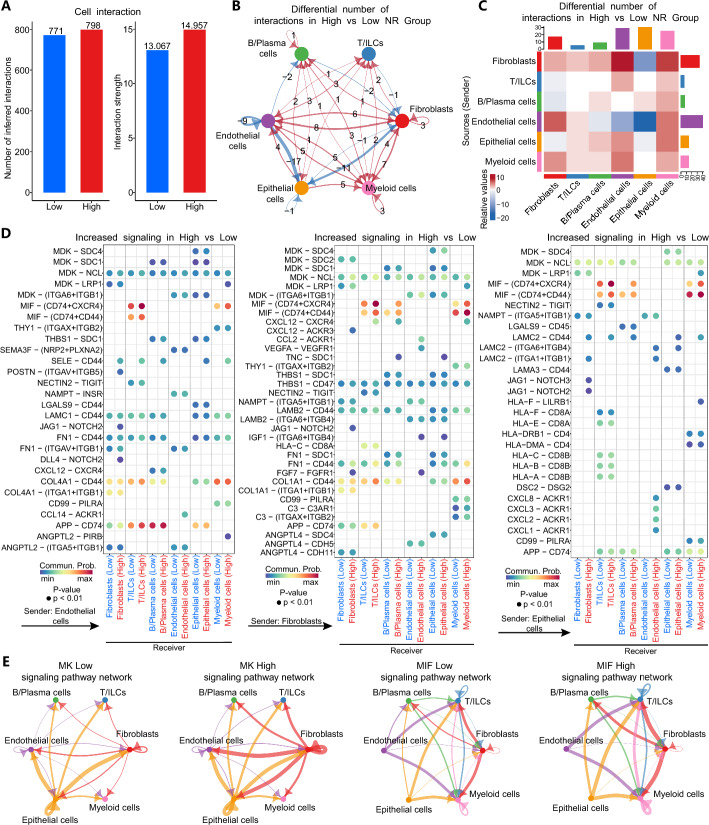
Variations in intercellular communications among different NR risk groups. **A** Bar plot displaying the number and strength of intercellular interactions between the NR^low^ and NR^high^ group. **B** Circle plot displaying the interactions of cell types between different NR groups. **C** Heatmap depicting cellular interactions across various cell types, with incoming signaling shown by the colored bar plot above and outgoing signaling shown by colored bar plot on the right. **D** Upregulated ligand-receptor pairs between endothelial cells, fibroblasts, as well as epithelial cells with other cell types in the NR^high^ group compared to the low group. **E** Circle plots displaying the representative ranked significant signal pathways between different NR groups

To further analyze the differences in intercellular communications across different NR groups, we examined the interactions among six major cell types. In comparison to NR^low^ group, the cell interactions were significantly elevated in NR^high^ group (Fig. 6A). Notably, we observed substantial differential interactions in both incoming and outgoing signals of endothelial cells and fibroblasts (Fig. 6B, C). The flow of information in the SPP1, CCL, and Midkine (MDK) pathways prominently increased at NR^high^ group compared to NR^low^ group (Fig. S8A).

Compared to NR^low^ group, enhanced interactions of the MDK-SDC4, MDK-NCL, MDK-LRP1, MIF-CD74/CXCR4, and MIF-CD74/CD44 pathways were observed in NR^high^ group, particularly between endothelial cells, fibroblasts, and epithelial cells compared to other cell types. (Fig. 6D; Fig. S8B). Across all cell types, MIF and MDK pathways were significantly enriched in the endothelial cells, fibroblasts, and epithelial cells from NR^high^ group (Fig. 6E). We observed elevated MDK expression, predominantly in Mal_C8_GAST and Mal_C9_APOA2, in NR^high^ group. Additionally, MIF and MDK were upregulated in endothelial cells, fibroblasts, and epithelial cells in NR^high^ group (Fig. S9A, B). These findings were further corroborated by bulk data analysis (Fig. S9C). In summary, high necroptosis activity significantly enhances specific intercellular communications among endothelial cells, fibroblasts, epithelial cells, and other cell types. Notably, the aberrant activation of the MDK and MIF interactions was particularly prominent in these enhanced cellular interactions, suggesting that they may serve as key molecular interactions for NR intercellular communications.

## Discussion

Necroptosis, a regulated form of cell death, has emerged as a key player in tumorigenesis, immune modulation, and therapeutic resistance across cancers [30–32]. While previous studies have explored the characteristics of necroptosis and its influence on the TME in GC, the molecular mechanisms underlying necroptosis at single-cell level remain poorly understood [6]. Our study represents the first comprehensive integration of bulk and single-cell transcriptomic data to systematically dissect necroptosis features in GC. In contrast to prior prognostic signatures derived solely from bulk data, our multi-omics approach not only yielded a robust prognostic model but, more importantly, enabled high-resolution dissection of the necroptosis-influenced TME. These analyses revealed specific cellular ecosystems, such as the expansion of Fib_C6_COL1A1 and EC_C5_CCL21 in NR^high^ samples, and identified the MDK and MIF pathways as key intercellular crosstalk. These findings provide a mechanistic layer of understanding that was previously unattainable, highlighting the power of single-cell resolution in uncovering novel therapeutic targets and resistance mechanisms associated with necroptosis.

Through CoxBoost and plsRCox algorithms, we identified a NR model (MMRN1, IGFBP7, CNTN1, HEYL, CARTPT, GPX3, and FNDC1), enabling precise risk stratification (high vs. low NR groups) and novel survival prediction in GC patients from the TCGA database [20]. To validate the predictive efficacy of the NR model, we validated it in the GEO database. Additionally, we developed a nomogram in TCGA, which demonstrated high accuracy in predicting GC patients’ survival, underscoring its potential for clinical translation.

Integrating bulk and single-cell transcriptomic analyses, we observed a significant increase of NR score in endothelial cells and fibroblasts. CAFs, known for their crucial role in ECM remodeling, were found to contribute to immune exclusion by constructing physical barriers to immune cell infiltration [33, 34]. Fibroblasts in NR^high^ group exhibited elevated transcription levels of fibrillar collagen, which were corroborated by bulk transcriptome data. Concurrently, hypoxia signaling pathway was upregulated in NR^high^ samples. Consistent with previous studies, hypoxia levels increased progressively from the tumor periphery to the core, while immune cells predominantly accumulated in oxygen-rich peripheral regions. It suggests that abnormal ECM deposition may impede immune cell infiltration, under the influence of necroptosis. Our results propose that therapeutic strategies targeting CAFs or local ECM component in NR^high^ patients may enhance immune cell infiltration into central tumor regions, thereby improving immune-mediated tumor cell targeting and killing.

It is noteworthy that while necroptosis is classically described as immunogenic cell death, the NR^high^ group in our study presents an immunosuppressive microenvironment. This seemingly paradoxical phenomenon can be explained by the context-dependency of necroptosis: under acute conditions, necroptosis can activate anti-tumor immunity; however, in the chronic tumor microenvironment, repeated episodes of necroptosis may lead to immune exhaustion, recruit immunosuppressive cells, and form a physical barrier through stromal remodeling, ultimately shaping an immunosuppressive microenvironment. This phenomenon suggested that the immunological outcome of necroptosis may depend on its intensity, duration, and microenvironmental context.

Comparative analysis of intercellular interactions among the endothelial cells, fibroblasts, and epithelial cells in NR^high^ group revealed significant enrichment of the MDK and MIF signaling pathways. MIF (macrophage migration inhibitory factor), initially characterized as a macrophage motility inhibitor [35, 36], has been implicated in diverse tumorigenic processes, including cell proliferation, immune evasion, and angiogenesis [37–40]. Notably, MIF inhibitors have shown promise in combination therapies for various cancers [41–43]. Consistent with these reports, we observed elevated MIF expression in NR^high^ group. Crucially, cell–cell communication analysis demonstrated enriched MIF receptor-ligand pairs in epithelial cells, fibroblasts, and endothelial cells interactions with myeloid cells. These findings collectively suggest that necroptosis may modulate myeloid cell migration dynamics through MIF-mediated signaling.

MDK, a heparin-binding growth factor, plays a pivotal role in cellular growth, migration, angiogenesis, and differentiation [44, 45]. In this study, MDK expression was elevated in NR^high^ group, with pronounced upregulation in single-cell clusters (Mal_C8_GAST and Mal_C9_APOA2). The MDK-NCL receptor-ligand interaction was enriched in endothelial cells, fibroblasts and epithelial cells during their crosstalk with other cell types. Previous studies have established the MDK-NCL axis as a key regulator of immune modulation and tumor progression [46–48]. Specifically, MDK has been shown to drive tumor progression and activate EGFR signaling under hypoxia through its interaction with NCL [44, 49–51]. Based on these findings, we hypothesize that the Mal_C9_APOA2 may suppress immune cell infiltration in the TME via the MDK-NCL signaling pathway. Collectively, our results suggest that the MDK-NCL pathway, in concert with MIF-mediated crosstalk among endothelial cells, fibroblasts, epithelial cells, and other cellular components, may shape an immunosuppressive TME phenotype in NR^high^ patients. This is achieved by establishing physical (ECM remodeling) barriers at the tumor-stroma interface, ultimately restricting immune cell infiltration and promoting tumor immune evasion.

Several limitations should be noted. First, the entire analytical framework was constructed using public databases (TCGA and GEO). Although our model demonstrated good performance when validated using the GEO dataset, the absence of validation in prospective, multi-center independent cohorts may limit the clinical generalizability of the necroptosis-related prognostic model. Second, the core findings of this study, including the prognostic significance of the model genes, the functional roles of specific fibroblast and endothelial subpopulations, and the inferred intercellular communication networks, were primarily derived from computational analyses and bioinformatic predictions. They lacked direct functional experiments to confirm the underlying causal mechanisms. Third, although the scRNA-seq analysis provided valuable insights into cellular heterogeneity, the limited sample size and inherent technical constraints mean that the inferred cell–cell interactions lacked spatial information.

To address these limitations, future work will prioritize the collection of multi-center clinical cohorts with comprehensive treatment information to validate the prognostic value of the necroptosis-related signature. Functional validation experiments will also be emphasized, including experimental manipulation of key genes in gastric cancer cell lines, fibroblasts, and endothelial cells and the use of mouse models to clarify their roles in necroptosis, TME remodeling, and therapy resistance. Finally, spatial technologies will be employed to validate the predicted intercellular interactions within the TME.

## Conclusion

In conclusion, our integrative multi-omics analysis of bulk and single-cell transcriptomic data demonstrates that necroptosis plays a pivotal role in remodeling the GC microenvironment by modulating immune cell infiltration, cancer stemness, and molecular pathways, ultimately contributing to poor clinical outcomes. We established an NR prognostic model exhibiting excellent predictive performance for patient survival, highlighting its potential clinical utility for risk stratification and treatment decision-making. Several limitations of this study should be acknowledged. First, the core findings, including the prognostic significance of the model genes, the functional roles of specific fibroblast and endothelial subpopulations, and the inferred intercellular communication networks, were derived from computational analyses and lack direct functional experimental validation. Second, although the scRNA-seq analysis provided valuable insights into cellular heterogeneity, the sample size (26 patients) is relatively limited, which may limit the generalizability of our findings. Third, the entire analytical framework was constructed using public databases (TCGA and GEO), and the independent, prospective, multi-center validation cohorts are needed to confirm the clinical utility of the prognostic model.

## Supplementary Information

Additional file 1. Fig. S1. The flowchart of the study.

Additional file 2. Fig. S2. Landscape of genetic variations in NR genes across GC samples. **A** Mutation frequency of the top 20 NR genes in GC patients. **B** Comprehensive mutation landscape of GC samples. **C** Survival analyses of the OS-related genes. **D** Chromosomal distribution of CNV variation in OS-related genes. **E** CNV alterations of OS-related genes. **F** Comparative analysis of OS-related genes expression levels between normal and tumor samples.

Additional file 3. Fig. S3. Biological characteristics of both tumor clusters. **A**, **B** Consensus cumulative distribution function (CDF) plot (**A**) and area under CDF (**B**) of unsupervised consensus clustering of OS-related genes. **C** The t-SNE analysis showing the distribution of patients. **D** The differential expression levels of immune checkpoint, T cell, M2 macrophage, cytotoxic, and resident markers between the two tumor clusters. **E**, **F** Spearman correlation between OS-related genes expression and immune infiltration calculated by CIBERSORT analysis and ssGSEA in GSE84437 (**E**) and GSE26942 (**F**) datasets. **G** The proportion of potential immunotherapy in the two tumor clusters evaluated by TIDE analysis.

Additional file 4. Fig. S4. Test of the NR prognostic model’s predictive accuracy. **A** KM curves showing OS of GC patients based on the expressions of MMRN1, IGFBP7, CNTN1, HEYL, CARTPT, GPX3, and FNDC1. **B**, **C** Scatter plots of PCA scores and t-SNE visualization of NR groups in the TCGA dataset (**B**) and GEO dataset (**C**). **D** Boxplot showing the distribution of NR scores across different tumor stages and T stages. **E** ROC curves demonstrating OS prediction of GC patients in TCGA and GSE84437 based on NR scores. **F** Calibration plots showing the accuracy of the nomogram in predicting the OS. **G** The DCA for the predictive value of the NR nomogram and other clinical features. **H** Violin plots illustrating the distribution of IC50 values for drugs between NR^low^ and NR^high^ groups. **I** The GSVA results demonstrating differential enrichment patterns of hallmark gene sets between NR^low^ and NR^high^ groups.

Additional file 5. Fig. S5. Distinct endothelial cell states in different NR status. **A** The t-SNE plot illustrating single cell profiles colored from distinct GC patients. **B** The t-SNE plot displaying expression profiles of the annotated genes. **C** The relative proportions of cell types in GC patients. **D** Dot plot displaying the expression levels of DEGs across all endothelial cell subtypes. **E** Dot plot displaying expression level of model genes across distinct endothelial subclusters. **F** Composition of EC subtypes in different NR groups. **G** Correlation between EC_C5_CCL21 abundance and NR score in GC samples (TCGA dataset).

Additional file 6. Fig. S6. Composition of fibroblasts in GC. **A** The t-SNE plots displaying the distribution of GC patients with the fibroblasts. **B** Dot plot displaying the expression of markers across fibroblast subtypes. **C** The relative proportion distribution of fibroblast subtypes across various GC patients. **D** Dot plot showing the expression patterns of model genes. **E** Univariate Cox regression analysis for OS based on markers from the fibroblast subtypes in the TCGA cohort. **F** KM curves illustrating the OS in GC samples, stratified by fibroblast subtypes abundance. **G** Positive correlation of Fib_C6_COL1A1 subset with NR score. **H** GO pathway enrichment analysis of DEGs between the two NR groups in all fibroblasts.

Additional file 7. Fig. S7. Heterogeneity of epithelial cells in GC. **A** Circle plots illustrating the differential intercellular communication networks between NR^low^ and NR^high^ tumors, with increased signaling shown in red colored edges and decreased signaling in blue colored edges (labeled by number). **B** The t-SNE plot of epithelial cells colored by their origin. **C** Expression patterns of genes characterizing distinct epithelial cell subtypes. **D** Heatmap visualization showing large-scale CNVs in single cells, inferred from scRNA-seq data, with T/ILCs, and myeloid cells as reference. **E** KM curves illustrating the OS based on epithelial cell subtypes of GC patients from the TCGA cohort. **F** Bar plot showing enriched GO terms of upregulated genes from Mal_C9_APOA2 in NR^high^ group.

Additional file 8. Fig. S8. Cell-cell interactions across various cell types. **A** Bar plots illustrating ligand-receptors signaling pathways based on differences in both NR groups. **B** Comparison of upregulated ligand-receptor pairs in endothelial cells, fibroblasts, as well as epithelial cells between the NR^low^ and NR^high^ group.

Additional file 9. Fig. S9. Differential expression of MIF and MDK signaling ligand-receptor pairs in both NR groups. **A** Dot plots showing gene expression patterns within MIF and MDK signaling networks across various cell subtypes in NR^low^ and NR^high^ group. **B** Dot plots displaying the expression of MIF and MDK signaling ligand-receptor pairs in both NR groups. **C** Violin plots illustrating the expression levels of MIF- and MDK-related genes between the two NR groups in bulk RNA-seq data.

Additional file 10 (XLSX 15 KB)

Additional file 11 (PDF 62 KB)

## Data Availability

The datasets analyzed during the current study are available in public, open-access repositories listed in this article. The datasets we analyzed during the current study are available in the Gene Expression Omnibus (GEO; https:/www.ncbi.nlm.nih.gov/geo): GSE183904, GSE84437 and GSE26942; The Cancer Genome Atlas (TCGA-STAD; https:/portal.gdc.cancer.gov); The Molecular Signatures Database (MSigDB; https:/www.gsea-msigdb.org/gsea/msigdb), the search keyword is “Necroptosis”; Kyoto Encyclopedia of Genes and Genomes (KEGG-map04217; http:/www.genome.jp/kegg); CancerSEA database (http:/biocc.hrbmu.edu.cn/CancerSEA; Stemness); Search Tool for the Retrieval of Interacting Genes database (STRING; https:/string-db.org).

## Abbreviations

GC: Gastric cancer
OS: Overall survival
DFI: Disease free interval
PFI: Progression free interval
DSS: Disease specific survival
C-index: C-concordance index
TCGA: The Cancer Genome Atlas
GEO: Gene Expression Omnibus
PPI: Protein-protein interaction
TMB: Tumor mutational burden
ssGSEA: Single sample gene set enrichment analysis
GSVA: Gene set variation analysis
KEGG: Kyoto encyclopedia of genes and genomes
GO: Gene ontology
GSEA: Gene set enrichment analysis
PCA: Principal component analysis
TME: Tumor microenvironment
ROC: Receiver operating characteristic
AUC: Area under the time-dependent ROC curve
IC50: Half-maximal inhibitory concentration
TIME: Tumor immune microenvironment
ICB: Immune checkpoint blockade
t-SNE: T-distributed stochastic neighbor embedding
ECs: Endothelial cells
ICBs: Immune checkpoint blockades
IPS: Immunophenoscore
scRNA-seq: Single-cell RNA sequencing
NR: Necroptosis-related
mRNA: Messenger RNA
DEGs: Differentially expressed genes
FDR: False discovery rate
FC: Fold change
KM: Kaplan-Meier
DCA: Decision curve analysis
DNN: Deep neural network
HVGs: Highly variable genes
ILCs: Innate lymphoid cells
HMM: Hidden Markov model
CNV: Copy number variation
MCPcounter: Microenvironment cell populations counter
LASSO: Least absolute shrinkage and selection operator
SuperPC: Supervised principal components
Enet: Elastic network
GBM: Gradient boosting machine
plsRcox: Partial least squares regression for Cox
RSF: Random forest
Survival-SVM: Survival support vector machine
GTEx: The Genotype-Tissue Expression
STAD: Stomach adenocarcinoma
OR: Odds ratios
