# Supplementary material for: Integrated single-cell and bulk transcriptomic analyses unveil a necroptosis-related prognostic model and its association with tumor microenvironment remodeling in gastric cancer
Source: Discov Oncol. 2026 May 16;17:1125. doi: 10.1007/s12672-026-05109-7 (PMC13442796; doi:10.1007/s12672-026-05109-7)
Supplement: Supplementary file 1 [file 12672_2026_5109_MOESM11_ESM.pdf]

Supplementary Figures

Fig S1. The flowchart of the study.

Fig S2. Landscape of genetic variations in NR genes across GC samples.

A Mutation frequency of the top 20 NR genes in GC patients. **B** Comprehensive mutation landscape of GC samples. **C** Survival analyses of the OS-related genes. **D** Chromosomal distribution of CNV variation in OS-related genes. **E** CNV alterations of OS-related genes. **F** Comparative analysis of OS-related genes expression levels between normal and tumor samples.

Fig S3. Biological characteristics of both tumor clusters.

A, B Consensus cumulative distribution function (CDF) plot **(A)** and area under CDF **(B)** of unsupervised consensus clustering of OS-related genes. **C** The t-SNE analysis showing the distribution of patients. **D** The differential expression levels of immune checkpoint, T cell, M2 macrophage, cytotoxic, and resident markers between the two tumor clusters. **E, F** Spearman correlation between OS-related genes expression and immune infiltration calculated by CIBERSORT analysis and ssGSEA in GSE84437 **(E)** and GSE26942 **(F)** datasets. **G** The proportion of potential immunotherapy in the two tumor clusters evaluated by TIDE analysis.

Fig S4. Test of the NR prognostic model's predictive accuracy.

A KM curves showing OS of GC patients based on the expressions of MMRN1, IGFBP7, CNTN1, HEYL, CARTPT, GPX3, and FNDC1. **B, C** Scatter plots of PCA scores and t-SNE visualization of NR groups in the TCGA dataset **(B)** and GEO dataset **(C). D** Boxplot showing the distribution of NR scores across different tumor stages and T stages. **E** ROC curves demonstrating OS prediction of GC patients in TCGA and GSE84437 based on NR scores. **F** Calibration plots showing the accuracy of the nomogram in predicting the OS. **G** The DCA for the predictive value of the NR nomogram and other clinical features. **H** Violin plots illustrating the distribution of IC50 values for drugs between NR^{low} and NR^{high} groups. **I** The GSVA results demonstrating differential enrichment patterns of hallmark gene sets between NR^{low} and NR^{high} groups.

Fig S5. Distinct endothelial cell states in different NR status.

A The t-SNE plot illustrating single cell profiles colored from distinct GC patients. **B** The t-SNE plot displaying expression profiles of the annotated genes. **C** The relative proportions of cell types in GC patients. **D** Dot plot displaying the expression levels of DEGs across all endothelial cell subtypes. **E** Dot plot displaying expression level of model genes across distinct endothelial subclusters. **F** Composition of EC subtypes in

different NR groups. **G** Correlation between EC_C5_CCL21 abundance and NR score in GC samples (TCGA dataset).

Fig S6. Composition of fibroblasts in GC.

A The t-SNE plots displaying the distribution of GC patients with the fibroblasts. **B** Dot plot displaying the expression of markers across fibroblast subtypes. **C** The relative proportion distribution of fibroblast subtypes across various GC patients. **D** Dot plot showing the expression patterns of model genes. **E** Univariate Cox regression analysis for OS based on markers from the fibroblast subtypes in the TCGA cohort. **F** KM curves illustrating the OS in GC samples, stratified by fibroblast subtypes abundance. **G** Positive correlation of Fib_C6_COL1A1 subset with NR score. **H** GO pathway enrichment analysis of DEGs between the two NR groups in all fibroblasts.

Fig S7. Heterogeneity of epithelial cells in GC.

A Circle plots illustrating the differential intercellular communication networks between NR^{low} and NR^{high} tumors, with increased signaling shown in red colored edges and decreased signaling in blue colored edges (labeled by number). **B** The t-SNE plot of epithelial cells colored by their origin. **C** Expression patterns of genes characterizing distinct epithelial cell subtypes. **D** Heatmap visualization showing large-scale CNVs in single cells, inferred from scRNA-seq data, with T/ILCs, and myeloid cells as reference.

E KM curves illustrating the OS based on epithelial cell subtypes of GC patients from the TCGA cohort. **F** Bar plot showing enriched GO terms of upregulated genes from Mal_C9_APOA2 in NR^{high} group.

Fig S8. Cell-cell interactions across various cell types.

A Bar plots illustrating ligand-receptors signaling pathways based on differences in both NR groups. **B** Comparison of upregulated ligand-receptor pairs in endothelial cells, fibroblasts, as well as epithelial cells between the NR^{low} and NR^{high} group.

Fig S9. Differential expression of MIF and MDK signaling ligand-receptor pairs in both NR groups.

A Dot plots showing gene expression patterns within MIF and MDK signaling networks across various cell subtypes in NR^{low} and NR^{high} group. **B** Dot plots displaying the expression of MIF and MDK signaling ligand-receptor pairs in both NR groups. **C** Violin plots illustrating the expression levels of MIF- and MDK-related genes between the two NR groups in bulk RNA-seq data.
