# Supplementary material for: Integrated single-cell and bulk transcriptomic analyses unveil a necroptosis-related prognostic model and its association with tumor microenvironment remodeling in gastric cancer
Source: Discov Oncol. 2026 May 16;17:1125. doi: 10.1007/s12672-026-05109-7 (PMC13442796; doi:10.1007/s12672-026-05109-7)
Supplement: Supplementary file 2 [file 12672_2026_5109_MOESM1_ESM.pdf]

Step1: Identification of necroptosis-related subtypes

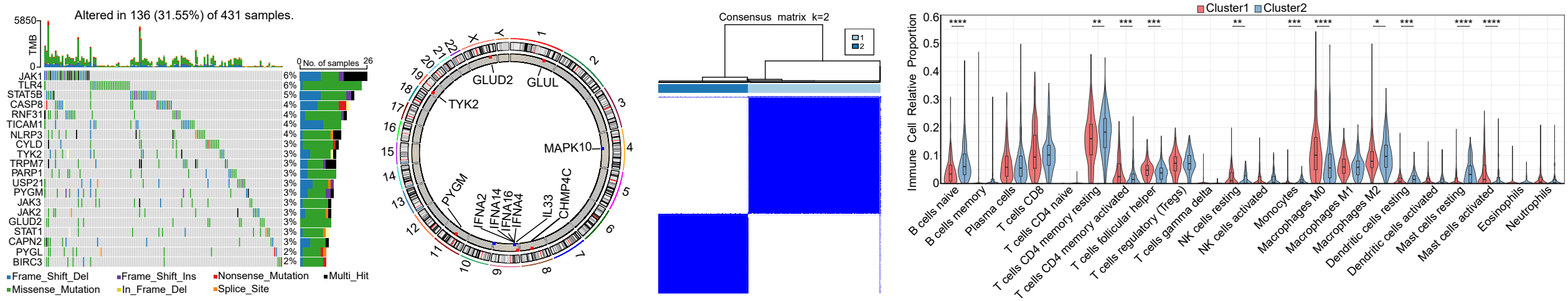

Bulk transcriptome analysis, differential analysis, clustering analysis, and immunological characterization

Step 2: Identification of a necroptosis-related scoring system

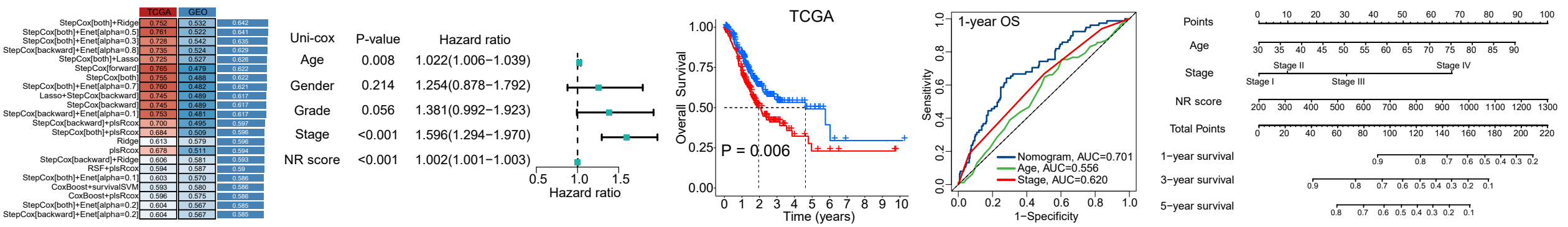

Establishment, validation, and Kaplan-Meier analysis of the diagnostic model

Step 3: Comparison of TME between disparate NR score groups

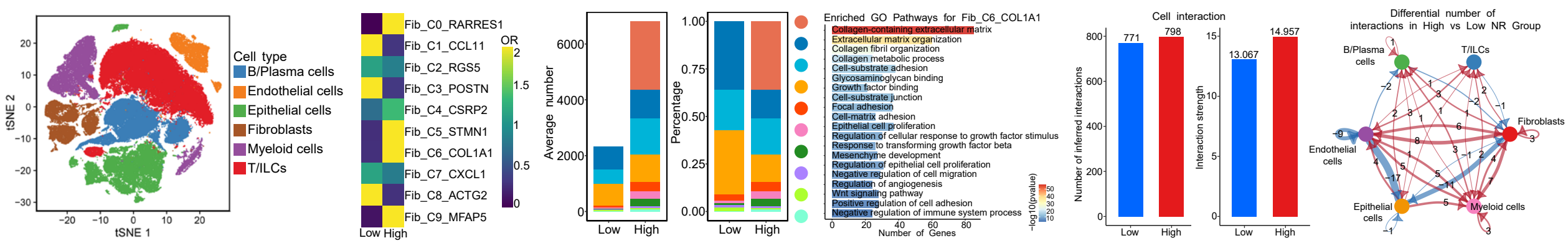

Functional analysis, cell-cell communication, and tumor stemness analysis at the bulk and single-cell levels
