# Supplementary material for: Integrated single-cell and bulk transcriptomic analyses unveil a necroptosis-related prognostic model and its association with tumor microenvironment remodeling in gastric cancer
Source: Discov Oncol. 2026 May 16;17:1125. doi: 10.1007/s12672-026-05109-7 (PMC13442796; doi:10.1007/s12672-026-05109-7)
Supplement: Supplementary file 3 [file 12672_2026_5109_MOESM2_ESM.pdf]

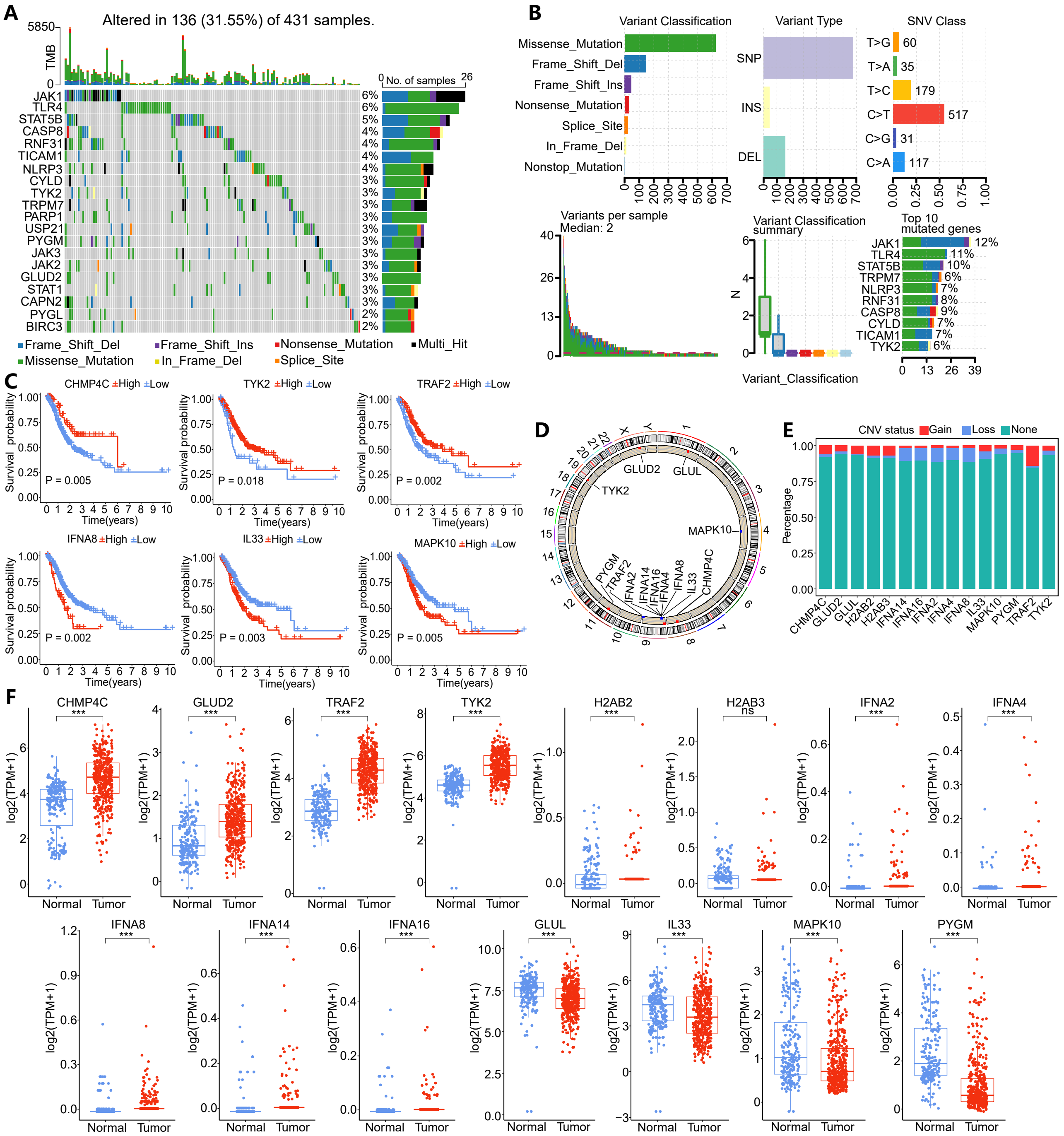
A
Altered in 136 (31.55%) of 431 samples.
TMB
5850
0
No. of samples
26
JAK1 6%
TLR4 6%
STAT5B 5%
CASP8 4%
RNF31 4%
TICAM1 4%
NLRP3 4%
CYLD 3%
TYK2 3%
TRPM7 3%
PARP1 3%
USP21 3%
PYGM 3%
JAK3 3%
JAK2 3%
GLUD2 3%
STAT1 3%
CAPN2 3%
PYGL 2%
BIRC3 2%
Frame_Shift_Del
Frame_Shift_Ins
Nonsense_Mutation
Multi_Hit
Missense_Mutation
In_Frame_Del
Splice_Site
B
Variant Classification
Missense_Mutation
Frame_Shift_Del
Frame_Shift_Ins
Nonsense_Mutation
Splice_Site
In_Frame_Del
Nonstop_Mutation
Variant Type
SNP
INS
DEL
SNV Class
T>G 60
T>A 35
T>C 179
C>T 517
C>G 31
C>A 117
Variants per sample
Median: 2
Variant Classification summary
N
Variant_Classification
Top 10 mutated genes
JAK1 12%
TLR4 11%
STAT5B 10%
TRPM7 6%
NLRP3 7%
RNF31 8%
CASP8 9%
CYLD 7%
TICAM1 7%
TYK2 6%
C
CHMP4C High Low
P = 0.005
TYK2 High Low
P = 0.018
TRAF2 High Low
P = 0.002
IFNA8 High Low
P = 0.002
IL33 High Low
P = 0.003
MAPK10 High Low
P = 0.005
Survival probability
Time(years)
D
GLUD2
GLUL
TYK2
MAPK10
PYGM
TRAF2
IFNA2
IFNA14
IFNA16
IFNA4
IFNA8
IL33
CHMP4C
E
CNV status Gain Loss None
Percentage
CHMP4C GLUD2 GLUL H2AB2 H2AB3 IFNA14 IFNA16 IFNA2 IFNA4 IFNA8 IL33 MAPK10 PYGM TRAF2 TYK2
F
CHMP4C ***
GLUD2 ***
TRAF2 ***
TYK2 ***
H2AB2 ***
H2AB3 ns
IFNA2 ***
IFNA4 ***
IFNA8 ***
IFNA14 ***
IFNA16 ***
GLUL ***
IL33 ***
MAPK10 ***
PYGM ***
log2(TPM+1)
Normal
Tumor
