# Supplementary material for: Integrated single-cell and bulk transcriptomic analyses unveil a necroptosis-related prognostic model and its association with tumor microenvironment remodeling in gastric cancer
Source: Discov Oncol. 2026 May 16;17:1125. doi: 10.1007/s12672-026-05109-7 (PMC13442796; doi:10.1007/s12672-026-05109-7)
Supplement: Supplementary file 4 [file 12672_2026_5109_MOESM3_ESM.pdf]

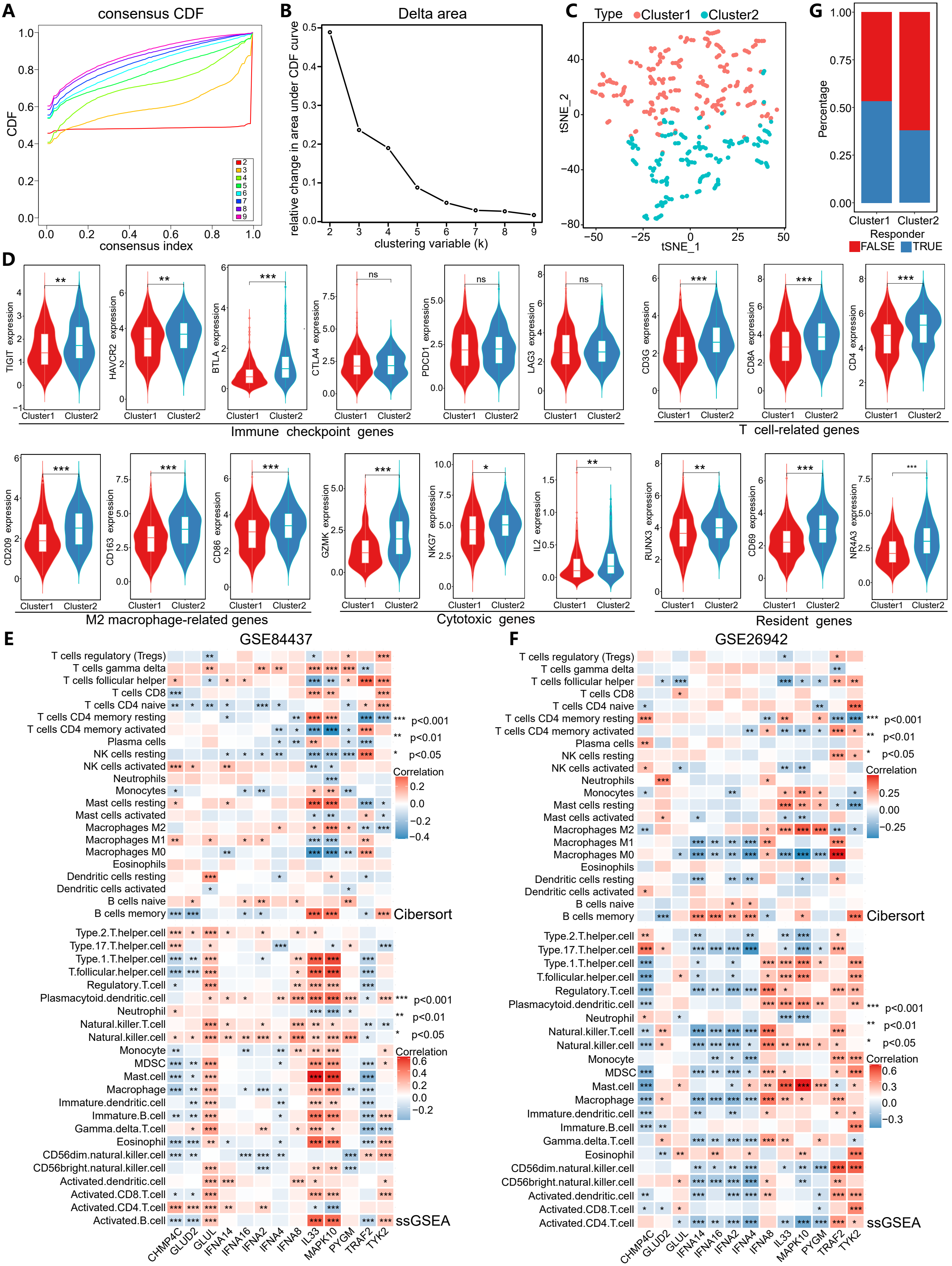

A
consensus CDF
CDF
consensus index
2
3
4
5
6
7
8
9
B
Delta area
relative change in area under CDF curve
clustering variable (k)
C
Type
Cluster1
Cluster2
tSNE_2
tSNE_1
G
Percentage
Cluster1
Cluster2
Responder
FALSE
TRUE
D
TIGIT expression
HAVCR2 expression
BTLA expression
CTLA4 expression
PDCD1 expression
LAG3 expression
CD3G expression
CD8A expression
CD4 expression
Immune checkpoint genes
T cell-related genes
CD209 expression
CD163 expression
CD86 expression
GZMK expression
NKG7 expression
IL2 expression
RUNX3 expression
CD69 expression
NR4A3 expression
M2 macrophage-related genes
Cytotoxic genes
Resident genes
ns
E
GSE84437
F
GSE26942
T cells regulatory (Tregs)
T cells gamma delta
T cells follicular helper
T cells CD8
T cells CD4 naive
T cells CD4 memory resting
T cells CD4 memory activated
Plasma cells
NK cells resting
NK cells activated
Neutrophils
Monocytes
Mast cells resting
Mast cells activated
Macrophages M2
Macrophages M1
Macrophages M0
Eosinophils
Dendritic cells resting
Dendritic cells activated
B cells naive
B cells memory
Cibersort
*** p<0.001
** p<0.01
* p<0.05
Correlation
Type.2.T.helper.cell
Type.17.T.helper.cell
Type.1.T.helper.cell
T.follicular.helper.cell
Regulatory.T.cell
Plasmacytoid.dendritic.cell
Neutrophil
Natural.killer.T.cell
Natural.killer.cell
Monocyte
MDSC
Mast.cell
Macrophage
Immature.dendritic.cell
Immature.B.cell
Gamma.delta.T.cell
Eosinophil
CD56dim.natural.killer.cell
CD56bright.natural.killer.cell
Activated.dendritic.cell
Activated.CD8.T.cell
Activated.CD4.T.cell
Activated.B.cell
ssGSEA
CHMP4C
GLUD2
GLUL
IFNA14
IFNA16
IFNA2
IFNA4
IFNA8
IL33
MAPK10
PYGM
TRAF2
TYK2
