# Supplementary material for: Integrated single-cell and bulk transcriptomic analyses unveil a necroptosis-related prognostic model and its association with tumor microenvironment remodeling in gastric cancer
Source: Discov Oncol. 2026 May 16;17:1125. doi: 10.1007/s12672-026-05109-7 (PMC13442796; doi:10.1007/s12672-026-05109-7)
Supplement: Supplementary file 5 [file 12672_2026_5109_MOESM4_ESM.pdf]

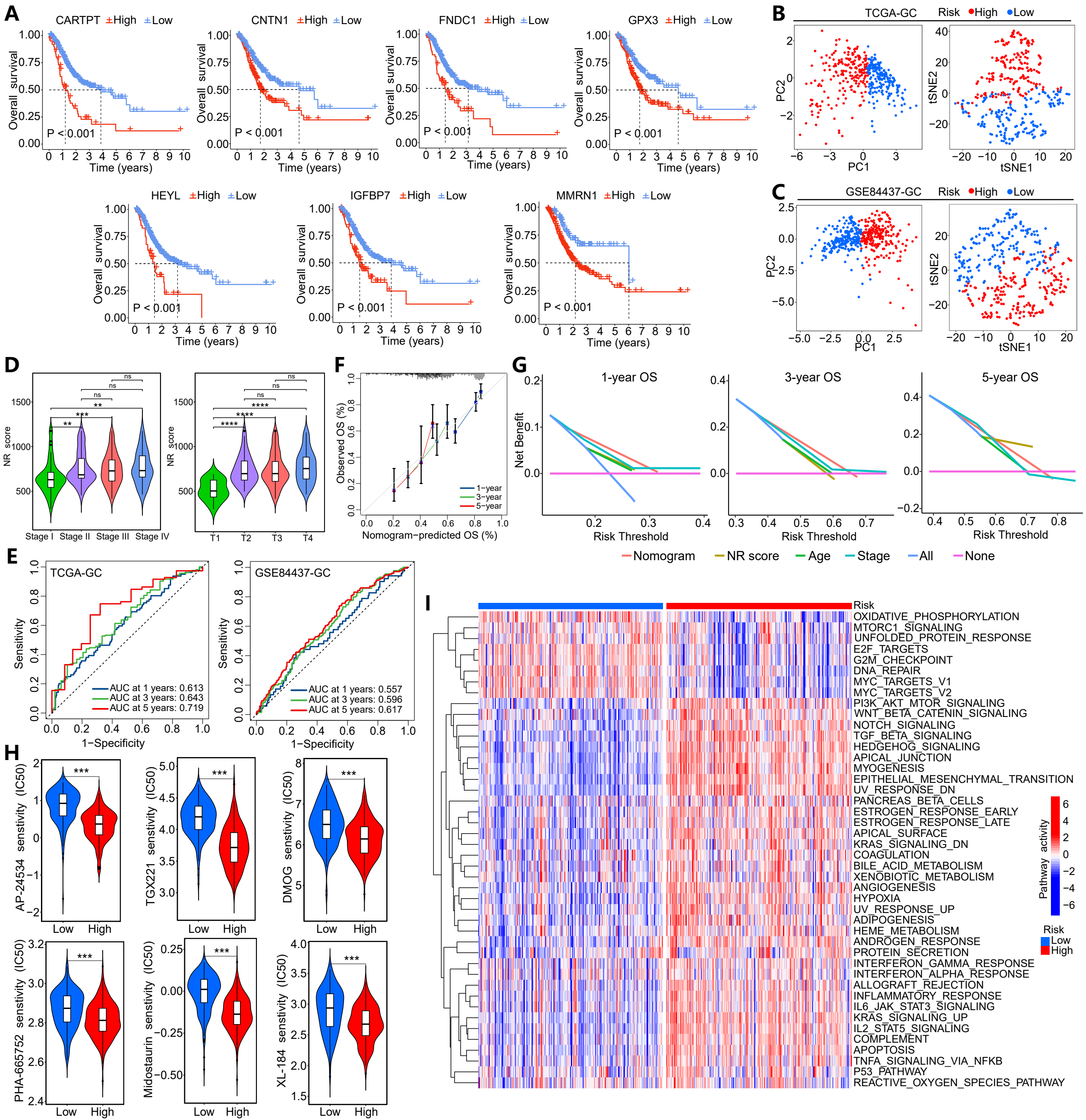
A
CARTPT ±High ±Low
CNTN1 ±High ±Low
FNDC1 ±High ±Low
GPX3 ±High ±Low
HEYL ±High ±Low
IGFBP7 ±High ±Low
MMRN1 ±High ±Low
Overall survival
Time (years)
P < 0.001
B
TCGA-GC Risk High Low
PC1
PC2
tSNE1
tSNE2
C
GSE84437-GC Risk High Low
D
NR score
Stage I Stage II Stage III Stage IV
T1 T2 T3 T4
ns
E
TCGA-GC
AUC at 1 years: 0.613
AUC at 3 years: 0.643
AUC at 5 years: 0.719
GSE84437-GC
AUC at 1 years: 0.557
AUC at 3 years: 0.596
AUC at 5 years: 0.617
Sensitivity
1−Specificity
F
Observed OS (%)
Nomogram−predicted OS (%)
1−year
3−year
5−year
G
1-year OS
3-year OS
5-year OS
Net Benefit
Risk Threshold
Nomogram NR score Age Stage All None
H
AP-24534 sensitivity (IC50)
TGX221 sensitivity (IC50)
DMOG sensitivity (IC50)
PHA-665752 sensitivity (IC50)
Midostaurin sensitivity (IC50)
XL-184 sensitivity (IC50)
Low High
I
Risk
OXIDATIVE_PHOSPHORYLATION
MTORC1_SIGNALING
UNFOLDED_PROTEIN_RESPONSE
E2F_TARGETS
G2M_CHECKPOINT
DNA_REPAIR
MYC_TARGETS_V1
MYC_TARGETS_V2
PI3K_AKT_MTOR_SIGNALING
WNT_BETA_CATENIN_SIGNALING
NOTCH_SIGNALING
TGF_BETA_SIGNALING
HEDGEHOG_SIGNALING
APICAL_JUNCTION
MYOGENESIS
EPITHELIAL_MESENCHYMAL_TRANSITION
UV_RESPONSE_DN
PANCREAS_BETA_CELLS
ESTROGEN_RESPONSE_EARLY
ESTROGEN_RESPONSE_LATE
APICAL_SURFACE
KRAS_SIGNALING_DN
COAGULATION
BILE_ACID_METABOLISM
XENOBIOTIC_METABOLISM
ANGIOGENESIS
HYPOXIA
UV_RESPONSE_UP
ADIPOGENESIS
HEME_METABOLISM
ANDROGEN_RESPONSE
PROTEIN_SECRETION
INTERFERON_GAMMA_RESPONSE
INTERFERON_ALPHA_RESPONSE
ALLOGRAFT_REJECTION
INFLAMMATORY_RESPONSE
IL6_JAK_STAT3_SIGNALING
KRAS_SIGNALING_UP
IL2_STAT5_SIGNALING
COMPLEMENT
APOPTOSIS
TNFA_SIGNALING_VIA_NFKB
P53_PATHWAY
REACTIVE_OXYGEN_SPECIES_PATHWAY
Pathway activity
Risk
Low
High
