# Supplementary material for: Integrated single-cell and bulk transcriptomic analyses unveil a necroptosis-related prognostic model and its association with tumor microenvironment remodeling in gastric cancer
Source: Discov Oncol. 2026 May 16;17:1125. doi: 10.1007/s12672-026-05109-7 (PMC13442796; doi:10.1007/s12672-026-05109-7)
Supplement: Supplementary file 6 [file 12672_2026_5109_MOESM5_ESM.pdf]

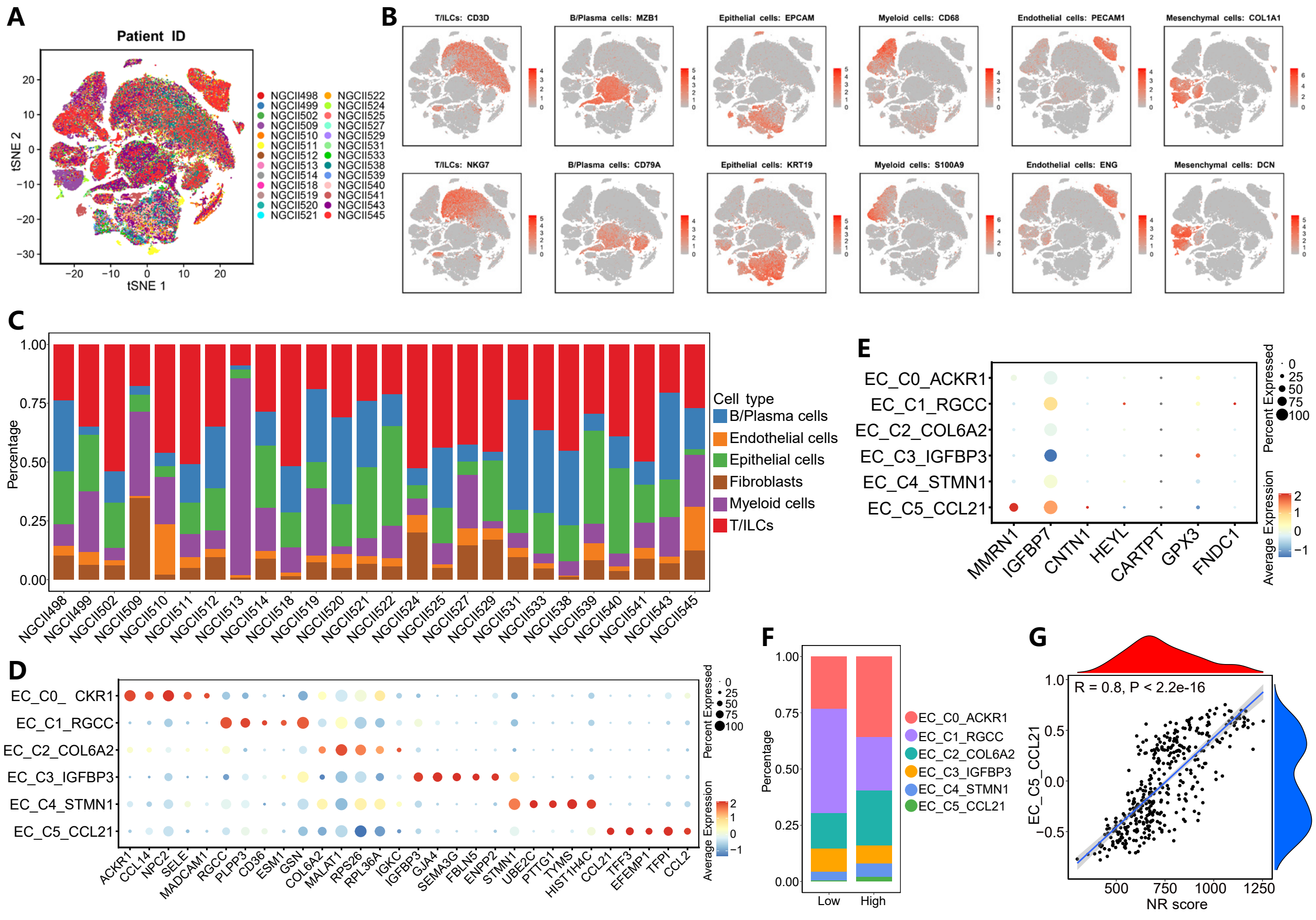

A
Patient ID
tSNE 1
tSNE 2
NGCII498 NGCII499 NGCII502 NGCII509 NGCII510 NGCII511 NGCII512 NGCII513 NGCII514 NGCII518 NGCII519 NGCII520 NGCII521
NGCII522 NGCII524 NGCII525 NGCII527 NGCII529 NGCII531 NGCII533 NGCII538 NGCII539 NGCII540 NGCII541 NGCII543 NGCII545
B
T/ILCs: CD3D
B/Plasma cells: MZB1
Epithelial cells: EPCAM
Myeloid cells: CD68
Endothelial cells: PECAM1
Mesenchymal cells: COL1A1
T/ILCs: NKG7
B/Plasma cells: CD79A
Epithelial cells: KRT19
Myeloid cells: S100A9
Endothelial cells: ENG
Mesenchymal cells: DCN
C
Percentage
Cell type
B/Plasma cells
Endothelial cells
Epithelial cells
Fibroblasts
Myeloid cells
T/ILCs
D
EC_C0_ CKR1
EC_C1_RGCC
EC_C2_COL6A2
EC_C3_IGFBP3
EC_C4_STMN1
EC_C5_CCL21
ACKR1 CCL14 NPC2 SELE MADCAM1 RGCC PLPP3 CD36 ESM1 GSN COL6A2 MALAT1 RPS26 RPL36A IGKC IGFBP3 GJA4 SEMA3G FBLN5 ENPP2 STMN1 UBE2C PTTG1 TYMS HIST1H4C CCL21 TFF3 EFEMP1 TFPI CCL2
Percent Expressed
Average Expression
E
EC_C0_ACKR1
EC_C1_RGCC
EC_C2_COL6A2
EC_C3_IGFBP3
EC_C4_STMN1
EC_C5_CCL21
MMRN1 IGFBP7 CNTN1 HEYL CARTPT GPX3 FNDC1
Percent Expressed
Average Expression
F
Percentage
Low
High
EC_C0_ACKR1
EC_C1_RGCC
EC_C2_COL6A2
EC_C3_IGFBP3
EC_C4_STMN1
EC_C5_CCL21
G
R = 0.8, P < 2.2e-16
EC_C5_CCL21
NR score
