# Supplementary material for: Integrated single-cell and bulk transcriptomic analyses unveil a necroptosis-related prognostic model and its association with tumor microenvironment remodeling in gastric cancer
Source: Discov Oncol. 2026 May 16;17:1125. doi: 10.1007/s12672-026-05109-7 (PMC13442796; doi:10.1007/s12672-026-05109-7)
Supplement: Supplementary file 7 [file 12672_2026_5109_MOESM6_ESM.pdf]

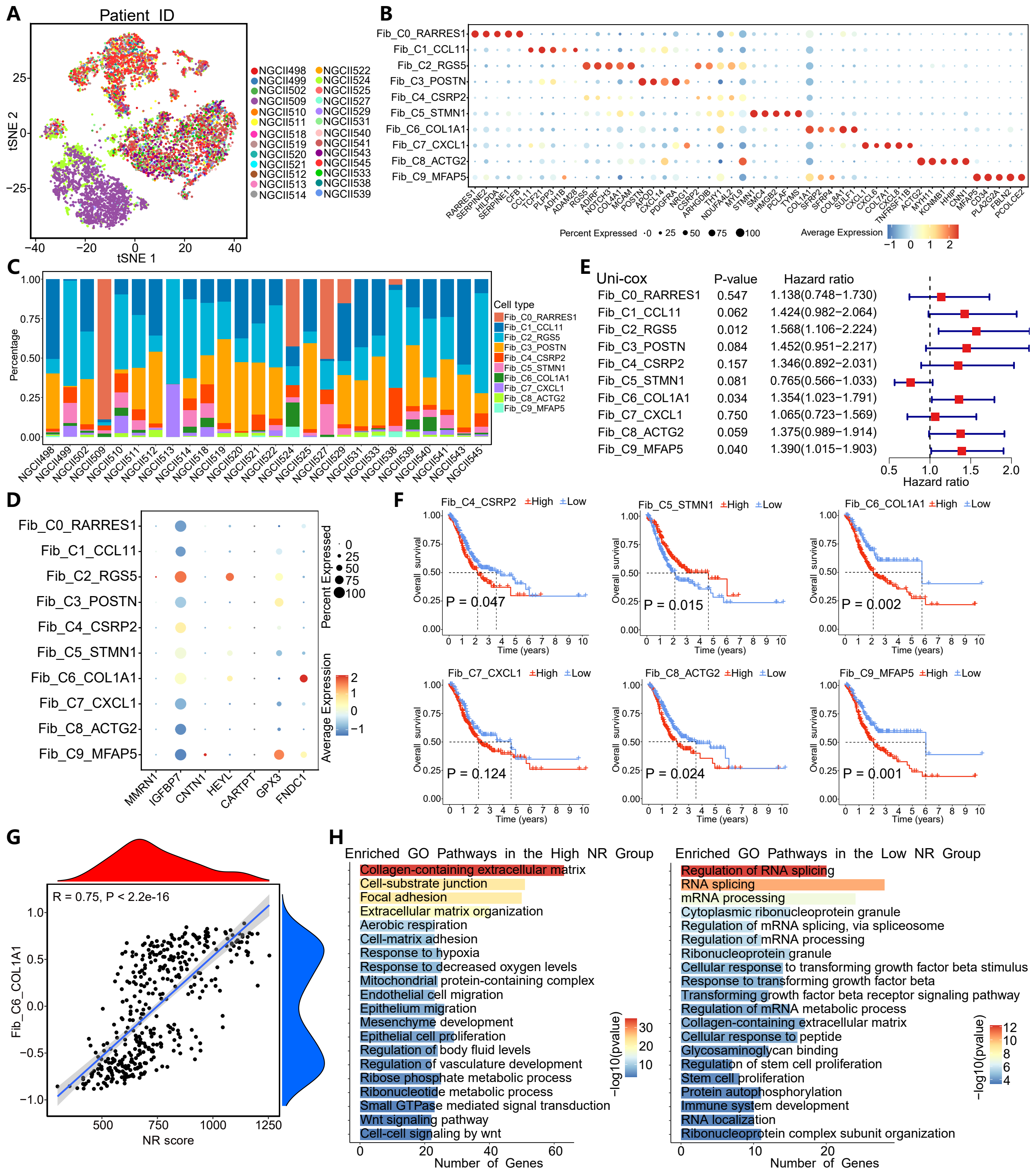
A
Patient ID
tSNE 1
tSNE 2
NGCII498
NGCII499
NGCII502
NGCII509
NGCII510
NGCII511
NGCII518
NGCII519
NGCII520
NGCII521
NGCII512
NGCII513
NGCII514
NGCII522
NGCII524
NGCII525
NGCII527
NGCII529
NGCII531
NGCII540
NGCII541
NGCII543
NGCII545
NGCII533
NGCII538
NGCII539
B
Fib_C0_RARRES1
Fib_C1_CCL11
Fib_C2_RGS5
Fib_C3_POSTN
Fib_C4_CSRP2
Fib_C5_STMN1
Fib_C6_COL1A1
Fib_C7_CXCL1
Fib_C8_ACTG2
Fib_C9_MFAP5
RARRES1 SERPINE2 HILPDA SERPINE1 CFB CCL11 TCF21 PLPP3 ADH1B ADAM28 RGS5 ADIRF NOTCH3 COL4A1 MCAM POSTN APOD CXCL14 PDGFRA NRG1 CSRP2 ARHGDIB THY1 NDUFA4L2 MYL9 STMN1 SMC4 HMGB2 PCLAF TYMS COL1A1 SFRP2 SFRP4 COL8A1 SULF1 CXCL6 COL7A1 CXCL8 TNFRSF11B ACTG2 MYH11 KCNMB1 HHIP CNN1 MFAP5 CD34 PLA2G2A FBLN2 PCOLCE2
Percent Expressed 0 25 50 75 100
Average Expression −1 0 1 2
C
Percentage
Cell type
Fib_C0_RARRES1
Fib_C1_CCL11
Fib_C2_RGS5
Fib_C3_POSTN
Fib_C4_CSRP2
Fib_C5_STMN1
Fib_C6_COL1A1
Fib_C7_CXCL1
Fib_C8_ACTG2
Fib_C9_MFAP5
NGCII498 NGCII499 NGCII502 NGCII509 NGCII510 NGCII511 NGCII512 NGCII513 NGCII514 NGCII518 NGCII519 NGCII520 NGCII521 NGCII522 NGCII524 NGCII525 NGCII527 NGCII529 NGCII531 NGCII533 NGCII538 NGCII539 NGCII540 NGCII541 NGCII543 NGCII545
E
Uni-cox P-value Hazard ratio
Fib_C0_RARRES1 0.547 1.138(0.748−1.730)
Fib_C1_CCL11 0.062 1.424(0.982−2.064)
Fib_C2_RGS5 0.012 1.568(1.106−2.224)
Fib_C3_POSTN 0.084 1.452(0.951−2.217)
Fib_C4_CSRP2 0.157 1.346(0.892−2.031)
Fib_C5_STMN1 0.081 0.765(0.566−1.033)
Fib_C6_COL1A1 0.034 1.354(1.023−1.791)
Fib_C7_CXCL1 0.750 1.065(0.723−1.569)
Fib_C8_ACTG2 0.059 1.375(0.989−1.914)
Fib_C9_MFAP5 0.040 1.390(1.015−1.903)
Hazard ratio
D
Fib_C0_RARRES1
Fib_C1_CCL11
Fib_C2_RGS5
Fib_C3_POSTN
Fib_C4_CSRP2
Fib_C5_STMN1
Fib_C6_COL1A1
Fib_C7_CXCL1
Fib_C8_ACTG2
Fib_C9_MFAP5
MMRN1 IGFBP7 CNTN1 HEYL CARTPT GPX3 FNDC1
Percent Expressed 0 25 50 75 100
Average Expression 2 1 0 −1
F
Fib_C4_CSRP2 High Low P = 0.047
Fib_C5_STMN1 High Low P = 0.015
Fib_C6_COL1A1 High Low P = 0.002
Fib_C7_CXCL1 High Low P = 0.124
Fib_C8_ACTG2 High Low P = 0.024
Fib_C9_MFAP5 High Low P = 0.001
Overall survival
Time (years)
G
R = 0.75, P < 2.2e-16
Fib_C6_COL1A1
NR score
H
Enriched GO Pathways in the High NR Group
Collagen-containing extracellular matrix
Cell-substrate junction
Focal adhesion
Extracellular matrix organization
Aerobic respiration
Cell-matrix adhesion
Response to hypoxia
Response to decreased oxygen levels
Mitochondrial protein-containing complex
Endothelial cell migration
Epithelium migration
Mesenchyme development
Epithelial cell proliferation
Regulation of body fluid levels
Regulation of vasculature development
Ribose phosphate metabolic process
Ribonucleotide metabolic process
Small GTPase mediated signal transduction
Wnt signaling pathway
Cell-cell signaling by wnt
-log10(pvalue)
Number of Genes
Enriched GO Pathways in the Low NR Group
Regulation of RNA splicing
RNA splicing
mRNA processing
Cytoplasmic ribonucleoprotein granule
Regulation of mRNA splicing, via spliceosome
Regulation of mRNA processing
Ribonucleoprotein granule
Cellular response to transforming growth factor beta stimulus
Response to transforming growth factor beta
Transforming growth factor beta receptor signaling pathway
Regulation of mRNA metabolic process
Collagen-containing extracellular matrix
Cellular response to peptide
Glycosaminoglycan binding
Regulation of stem cell proliferation
Stem cell proliferation
Protein autophosphorylation
Immune system development
RNA localization
Ribonucleoprotein complex subunit organization
-log10(pvalue)
Number of Genes
