# Supplementary material for: Integrated single-cell and bulk transcriptomic analyses unveil a necroptosis-related prognostic model and its association with tumor microenvironment remodeling in gastric cancer
Source: Discov Oncol. 2026 May 16;17:1125. doi: 10.1007/s12672-026-05109-7 (PMC13442796; doi:10.1007/s12672-026-05109-7)
Supplement: Supplementary file 8 [file 12672_2026_5109_MOESM7_ESM.pdf]

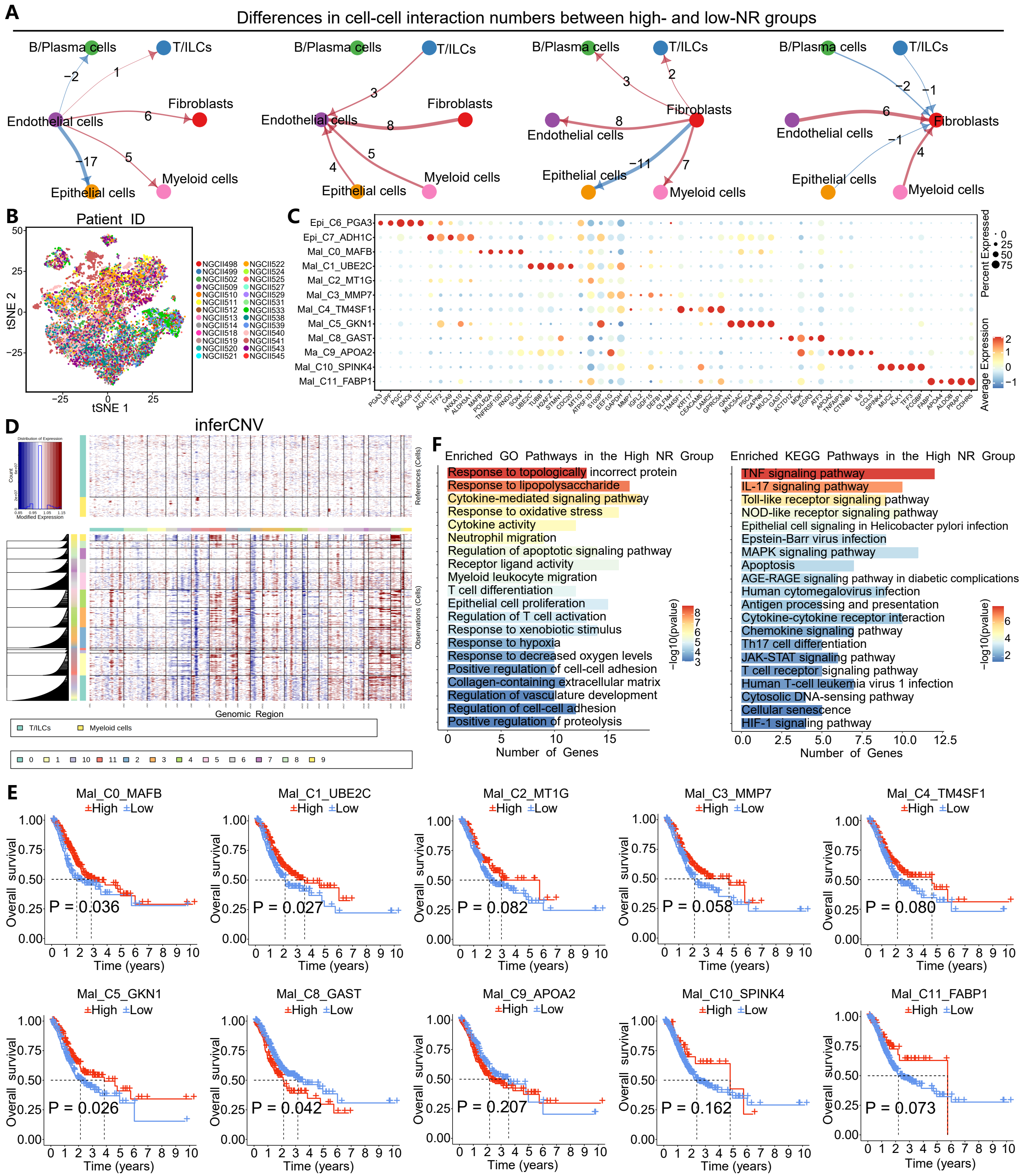
A
Differences in cell-cell interaction numbers between high- and low-NR groups
B/Plasma cells
T/ILCs
Fibroblasts
Endothelial cells
Epithelial cells
Myeloid cells
B
Patient ID
tSNE 1
tSNE 2
NGCII498
NGCII499
NGCII502
NGCII509
NGCII510
NGCII511
NGCII512
NGCII513
NGCII514
NGCII518
NGCII519
NGCII520
NGCII521
NGCII522
NGCII524
NGCII525
NGCII527
NGCII529
NGCII531
NGCII533
NGCII538
NGCII539
NGCII540
NGCII541
NGCII543
NGCII545
C
Epi_C6_PGA3
Epi_C7_ADH1C
Mal_C0_MAFB
Mal_C1_UBE2C
Mal_C2_MT1G
Mal_C3_MMP7
Mal_C4_TM4SF1
Mal_C5_GKN1
Mal_C8_GAST
Ma_C9_APOA2
Mal_C10_SPINK4
Mal_C11_FABP1
Percent Expressed
Average Expression
D
inferCNV
References (Cells)
Observations (Cells)
Genomic Region
T/ILCs
Myeloid cells
F
Enriched GO Pathways in the High NR Group
Response to topologically incorrect protein
Response to lipopolysaccharide
Cytokine-mediated signaling pathway
Response to oxidative stress
Cytokine activity
Neutrophil migration
Regulation of apoptotic signaling pathway
Receptor ligand activity
Myeloid leukocyte migration
T cell differentiation
Epithelial cell proliferation
Regulation of T cell activation
Response to xenobiotic stimulus
Response to hypoxia
Response to decreased oxygen levels
Positive regulation of cell-cell adhesion
Collagen-containing extracellular matrix
Regulation of vasculature development
Regulation of cell-cell adhesion
Positive regulation of proteolysis
Number of Genes
−log10(pvalue)
Enriched KEGG Pathways in the High NR Group
TNF signaling pathway
IL-17 signaling pathway
Toll-like receptor signaling pathway
NOD-like receptor signaling pathway
Epithelial cell signaling in Helicobacter pylori infection
Epstein-Barr virus infection
MAPK signaling pathway
Apoptosis
AGE-RAGE signaling pathway in diabetic complications
Human cytomegalovirus infection
Antigen processing and presentation
Cytokine-cytokine receptor interaction
Chemokine signaling pathway
Th17 cell differentiation
JAK-STAT signaling pathway
T cell receptor signaling pathway
Human T-cell leukemia virus 1 infection
Cytosolic DNA-sensing pathway
Cellular senescence
HIF-1 signaling pathway
Number of Genes
−log10(pvalue)
E
Mal_C0_MAFB P = 0.036
Mal_C1_UBE2C P = 0.027
Mal_C2_MT1G P = 0.082
Mal_C3_MMP7 P = 0.058
Mal_C4_TM4SF1 P = 0.080
Mal_C5_GKN1 P = 0.026
Mal_C8_GAST P = 0.042
Mal_C9_APOA2 P = 0.207
Mal_C10_SPINK4 P = 0.162
Mal_C11_FABP1 P = 0.073
High
Low
Overall survival
Time (years)
