# Supplementary material for: Integrated single-cell and bulk transcriptomic analyses unveil a necroptosis-related prognostic model and its association with tumor microenvironment remodeling in gastric cancer
Source: Discov Oncol. 2026 May 16;17:1125. doi: 10.1007/s12672-026-05109-7 (PMC13442796; doi:10.1007/s12672-026-05109-7)
Supplement: Supplementary file 9 [file 12672_2026_5109_MOESM8_ESM.pdf]

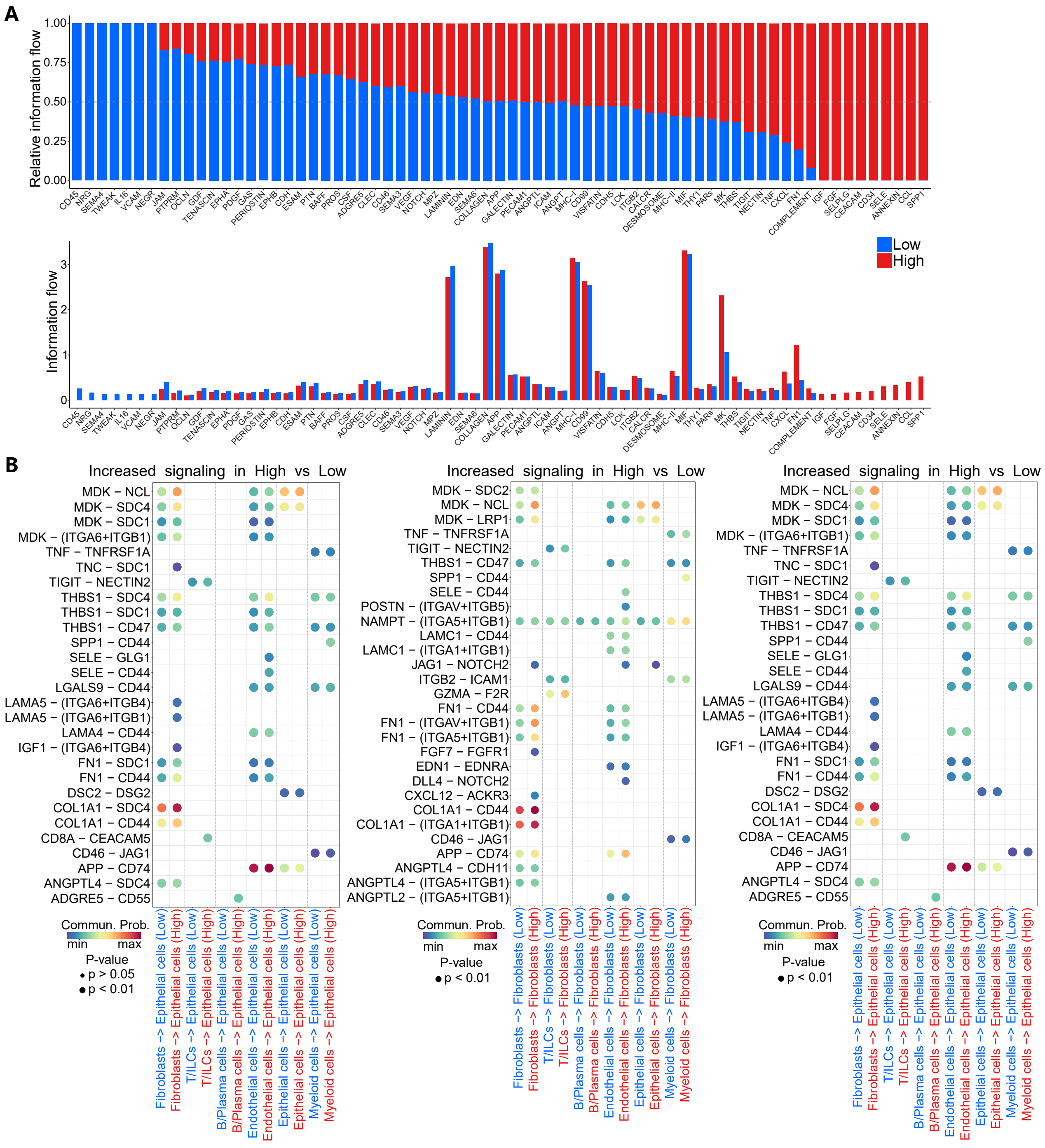

A
Relative information flow
1.00
0.75
0.50
0.25
0.00
CD45 NRG SEMA4 TWEAK IL16 VCAM NEGR JAM PTPRM OCLN GDF TENASCIN EPHA PDGF GAS PERIOSTIN EPHB CDH ESAM PTN BAFF PROS CSF ADGRE5 CLEC CD46 SEMA3 VEGF NOTCH MPZ LAMININ EDN SEMA6 COLLAGEN APP GALECTIN PECAM1 ANGPTL ICAM ANGPT MHC-I CD99 VISFATIN CDH5 LCK ITGB2 CALCR DESMOSOME MHC-II MIF THY1 PARs MK THBS TIGIT NECTIN TNF CXCL FN1 COMPLEMENT IGF FGF SELPLG CEACAM CD34 SELE ANNEXIN CCL SPP1
Information flow
3
2
1
0
Low
High
B
Increased signaling in High vs Low
MDK − NCL
MDK − SDC4
MDK − SDC1
MDK − (ITGA6+ITGB1)
TNF − TNFRSF1A
TNC − SDC1
TIGIT − NECTIN2
THBS1 − SDC4
THBS1 − SDC1
THBS1 − CD47
SPP1 − CD44
SELE − GLG1
SELE − CD44
LGALS9 − CD44
LAMA5 − (ITGA6+ITGB4)
LAMA5 − (ITGA6+ITGB1)
LAMA4 − CD44
IGF1 − (ITGA6+ITGB4)
FN1 − SDC1
FN1 − CD44
DSC2 − DSG2
COL1A1 − SDC4
COL1A1 − CD44
CD8A − CEACAM5
CD46 − JAG1
APP − CD74
ANGPTL4 − SDC4
ADGRE5 − CD55
Commun. Prob.
min max
P-value
p > 0.05
p < 0.01
Fibroblasts -> Epithelial cells (Low)
Fibroblasts -> Epithelial cells (High)
T/ILCs -> Epithelial cells (Low)
T/ILCs -> Epithelial cells (High)
B/Plasma cells -> Epithelial cells (Low)
B/Plasma cells -> Epithelial cells (High)
Endothelial cells -> Epithelial cells (Low)
Endothelial cells -> Epithelial cells (High)
Epithelial cells -> Epithelial cells (Low)
Epithelial cells -> Epithelial cells (High)
Myeloid cells -> Epithelial cells (Low)
Myeloid cells -> Epithelial cells (High)
Increased signaling in High vs Low
MDK − SDC2
MDK − NCL
MDK − LRP1
TNF − TNFRSF1A
TIGIT − NECTIN2
THBS1 − CD47
SPP1 − CD44
SELE − CD44
POSTN − (ITGAV+ITGB5)
NAMPT − (ITGA5+ITGB1)
LAMC1 − CD44
LAMC1 − (ITGA1+ITGB1)
JAG1 − NOTCH2
ITGB2 − ICAM1
GZMA − F2R
FN1 − CD44
FN1 − (ITGAV+ITGB1)
FN1 − (ITGA5+ITGB1)
FGF7 − FGFR1
EDN1 − EDNRA
DLL4 − NOTCH2
CXCL12 − ACKR3
COL1A1 − CD44
COL1A1 − (ITGA1+ITGB1)
CD46 − JAG1
APP − CD74
ANGPTL4 − CDH11
ANGPTL4 − (ITGA5+ITGB1)
ANGPTL2 − (ITGA5+ITGB1)
Commun. Prob.
min max
P-value
p < 0.01
Fibroblasts -> Fibroblasts (Low)
Fibroblasts -> Fibroblasts (High)
T/ILCs -> Fibroblasts (Low)
T/ILCs -> Fibroblasts (High)
B/Plasma cells -> Fibroblasts (Low)
B/Plasma cells -> Fibroblasts (High)
Endothelial cells -> Fibroblasts (Low)
Endothelial cells -> Fibroblasts (High)
Epithelial cells -> Fibroblasts (Low)
Epithelial cells -> Fibroblasts (High)
Myeloid cells -> Fibroblasts (Low)
Myeloid cells -> Fibroblasts (High)
Increased signaling in High vs Low
MDK − NCL
MDK − SDC4
MDK − SDC1
MDK − (ITGA6+ITGB1)
TNF − TNFRSF1A
TNC − SDC1
TIGIT − NECTIN2
THBS1 − SDC4
THBS1 − SDC1
THBS1 − CD47
SPP1 − CD44
SELE − GLG1
SELE − CD44
LGALS9 − CD44
LAMA5 − (ITGA6+ITGB4)
LAMA5 − (ITGA6+ITGB1)
LAMA4 − CD44
IGF1 − (ITGA6+ITGB4)
FN1 − SDC1
FN1 − CD44
DSC2 − DSG2
COL1A1 − SDC4
COL1A1 − CD44
CD8A − CEACAM5
CD46 − JAG1
APP − CD74
ANGPTL4 − SDC4
ADGRE5 − CD55
Commun. Prob.
min max
P-value
p < 0.01
Fibroblasts -> Epithelial cells (Low)
Fibroblasts -> Epithelial cells (High)
T/ILCs -> Epithelial cells (Low)
T/ILCs -> Epithelial cells (High)
B/Plasma cells -> Epithelial cells (Low)
B/Plasma cells -> Epithelial cells (High)
Endothelial cells -> Epithelial cells (Low)
Endothelial cells -> Epithelial cells (High)
Epithelial cells -> Epithelial cells (Low)
Epithelial cells -> Epithelial cells (High)
Myeloid cells -> Epithelial cells (Low)
Myeloid cells -> Epithelial cells (High)
