# Supplementary material for: Integrated single-cell and bulk transcriptomic analyses unveil a necroptosis-related prognostic model and its association with tumor microenvironment remodeling in gastric cancer
Source: Discov Oncol. 2026 May 16;17:1125. doi: 10.1007/s12672-026-05109-7 (PMC13442796; doi:10.1007/s12672-026-05109-7)
Supplement: Supplementary file 10 [file 12672_2026_5109_MOESM9_ESM.pdf]

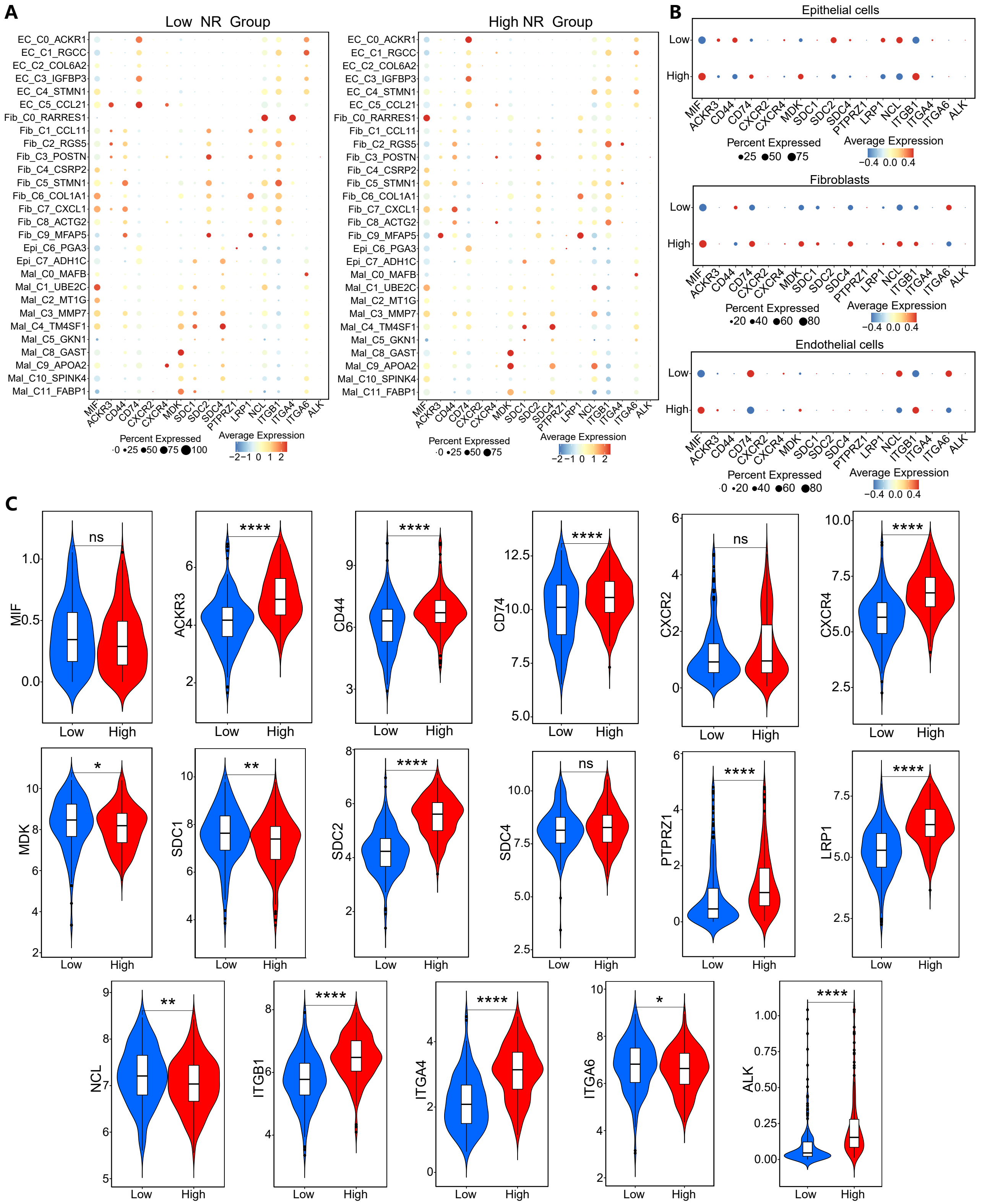
A
Low NR Group
High NR Group
EC_C0_ACKR1
EC_C1_RGCC
EC_C2_COL6A2
EC_C3_IGFBP3
EC_C4_STMN1
EC_C5_CCL21
Fib_C0_RARRES1
Fib_C1_CCL11
Fib_C2_RGS5
Fib_C3_POSTN
Fib_C4_CSRP2
Fib_C5_STMN1
Fib_C6_COL1A1
Fib_C7_CXCL1
Fib_C8_ACTG2
Fib_C9_MFAP5
Epi_C6_PGA3
Epi_C7_ADH1C
Mal_C0_MAFB
Mal_C1_UBE2C
Mal_C2_MT1G
Mal_C3_MMP7
Mal_C4_TM4SF1
Mal_C5_GKN1
Mal_C8_GAST
Mal_C9_APOA2
Mal_C10_SPINK4
Mal_C11_FABP1
MIF ACKR3 CD44 CD74 CXCR2 CXCR4 MDK SDC1 SDC2 SDC4 PTPRZ1 LRP1 NCL ITGB1 ITGA4 ITGA6 ALK
Percent Expressed
0 25 50 75 100
Average Expression
−2 −1 0 1 2
B
Epithelial cells
Fibroblasts
Endothelial cells
Low
High
Percent Expressed
25 50 75
20 40 60 80
0 20 40 60 80
Average Expression
−0.4 0.0 0.4
C
MIF ACKR3 CD44 CD74 CXCR2 CXCR4
MDK SDC1 SDC2 SDC4 PTPRZ1 LRP1
NCL ITGB1 ITGA4 ITGA6 ALK
ns
****
*
**
Low
High
